# Estimating the risk of brain metastasis for patients newly diagnosed with cancer

**DOI:** 10.1038/s43856-024-00445-7

**Published:** 2024-02-22

**Authors:** Joseph A. Miccio, Zizhong Tian, Sean S. Mahase, Christine Lin, Serah Choi, Brad E. Zacharia, Jason P. Sheehan, Paul D. Brown, Daniel M. Trifiletti, Joshua D. Palmer, Ming Wang, Nicholas G. Zaorsky

**Affiliations:** 1 Department of Radiation Oncology, Penn State Cancer Institute, Hershey, PA USA; 2 Division of Biostatistics and Bioinformatics, Department of Public Health Sciences, Penn State College of Medicine, Hershey, PA USA; 3 Department of Radiation Oncology, University Hospitals Seidman Cancer Center, Case Western Reserve School of Medicine, Cleveland, OH USA; 4 Department of Neurosurgery, Penn State Cancer Institute, Hershey, PA USA; 5 Department of Neurosurgery, University of Virginia School of Medicine, Charlottesville, VA USA; 6 Department of Radiation Oncology, Mayo Clinic, Rochester, MN USA; 7 Department of Radiation Oncology, Mayo Clinic, Jacksonville, FL USA; 8 Department of Radiation Oncology, The Ohio State University James Comprehensive Cancer Center, Columbus, OH USA; 9 Department of Population and Quantitative Health Sciences, Case Western Reserve University School of Medicine, Cleveland, OH USA

**Keywords:** CNS cancer, Cancer epidemiology, Cancer screening

## Abstract

**Background:**

Brain metastases (BM) affect clinical management and prognosis but limited resources exist to estimate BM risk in newly diagnosed cancer patients. Additionally, guidelines for brain MRI screening are limited. We aimed to develop and validate models to predict risk of BM at diagnosis for the most common cancer types that spread to the brain.

**Methods:**

Breast cancer, melanoma, kidney cancer, colorectal cancer (CRC), small cell lung cancer (SCLC), and non-small cell lung cancer (NSCLC) data were extracted from the National Cancer Database to evaluate for the variables associated with the presence of BM at diagnosis. Multivariable logistic regression (LR) models were developed and performance was evaluated with Area Under the Receiver Operating Characteristic Curve (AUC) and random-split training and testing datasets. Nomograms and a Webtool were created for each cancer type.

**Results:**

We identify 4,828,305 patients from 2010-2018 (2,095,339 breast cancer, 472,611 melanoma, 407,627 kidney cancer, 627,090 CRC, 164,864 SCLC, and 1,060,774 NSCLC). The proportion of patients with BM at diagnosis is 0.3%, 1.5%, 1.3%, 0.3%, 16.0%, and 10.3% for breast cancer, melanoma, kidney cancer, CRC, SCLC, and NSCLC, respectively. The average AUC over 100 random splitting for the LR models is 0.9534 for breast cancer, 0.9420 for melanoma, 0.8785 for CRC, 0.9054 for kidney cancer, 0.7759 for NSCLC, and 0.6180 for SCLC.

**Conclusions:**

We develop accurate models that predict the BM risk at diagnosis for multiple cancer types. The nomograms and Webtool may aid clinicians in considering brain MRI at the time of initial cancer diagnosis.

## Abstract (web summary)

Miccio et al. utilize the US National Cancer Database (NCDB) to develop models to estimate the risk of brain metastasis for newly-diagnosed cancers. They provide nomograms and a web tool for risk estimation across multiple cancer types.

## Plain language summary

When patients are diagnosed with cancer, it is unknown which patients have a significant risk of cancer spread to the brain. Cancer spread to the brain is important to diagnose since it changes how patients are treated and affects their prognosis. This study used a large national database of patients diagnosed with cancer and studied the characteristics that were associated with cancer spread to the brain. The results can be used by doctors to assess the risk of cancer spread to the brain and determine which patients with cancer may benefit most from brain imaging.

## Introduction

Over 200,000 patients with cancer are diagnosed with brain metastases (BM) annually in the United States^1,2^. Furthermore, BM incidence rates are increasing in the context of advances in systemic therapy and ubiquity of magnetic resonance imaging (MRI). While multiple validated models exist to estimate survival in patients with BM^3–5^, there are a dearth of models focusing on the presence of BM at initial diagnosis. Identifying patients with BM is crucial to guiding their optimal multidisciplinary management^6^.

The National Comprehensive Cancer Network (NCCN) guidelines provide considerations for brain MRI in only select circumstances for small cell lung cancer (SCLC), non-small cell lung cancer (NSCLC), breast cancer, kidney cancer, colorectal cancer (CRC) and melanoma^7–13^. NCCN recommends brain MRI for all patients with SCLC and most patients with NSCLC. The NCCN has no recommendations for brain MRI in CRC, and it is only recommended for breast and kidney cancer if there are suspicious central nervous system (CNS) symptoms. For melanoma, NCCN recommends brain MRI for stage IV disease and states it can be considered for stage IIIB/C/D disease. Notably, there is limited evidence to support these recommendations.

Given the limitations of the current guidelines regarding brain MRI for patients with a new diagnosis of cancer, we aimed to develop and validate cancer-specific models to predict the presence of BM at time of cancer diagnosis. The results of this work will be helpful to the multidisciplinary team of physicians that care for patients with cancer who are at risk of harboring brain metastases. We were successful in creation and validation of these models which have a varying degree of accuracy between different cancer types.

## Methods

### Data

The National Cancer Database (NCDB) is a joint project of the Commission on Cancer of the American College of Surgeons and the American Cancer Society, which consists of de-identified information regarding patient demographics, tumor characteristics, first-course treatment for the corresponding diagnosis, and survival for ~70% of patients diagnosed with cancer within the United States^14^. The data used in the study are derived from a de-identified NCDB file. The American College of Surgeons and the Commission on Cancer have not verified and are not responsible for the analytic or statistical methodology employed, or the conclusions drawn from these data by the investigator. The data used in this study were derived from a de-identified file, and thus were exempt from institutional review. No informed consent is required when using NCDB data.

The NCDB 2010–2018 data including the demographic and clinical characteristics were used for analysis. The primary outcome was defined as the presence of BM at time of diagnosis. The summary statistics including mean with standard deviation for continuous variables and frequency with percentage for categorical variables were provided for overall and stratified by 6 cancer sites (breast cancer, melanoma, kidney cancer, CRC, SCLC, and NSCLC). Other cancers were excluded given the relatively low incidence of brain metastases^15,16^. The model fitting was performed for each cancer type by considering 10 common risk predictors (e.g., patient age, sex, race, tumor grade, clinical primary tumor stage (T stage), clinical nodal stage (N stage), presence of bone metastases, presence of lung metastases, and presence of liver metastases) and cancer-specific factors (including available tumor markers) that have been shown previously to be prognostic^17–24^. Notably, patients in our analysis may have had stage I-III disease (if they are coded to have no bone, lung, liver, or brain metastases), or stage IV disease with brain only metastases if they are coded to have brain metastases but no bone, lung, or liver metastases.

### Statistical analysis

Group comparisons between patients with and without BM were based on the Pearson Chi-square tests (for categorical variables) and the two-sample *t*-test as well as the Wilcoxon Rank Sum test (for the continuous variable, age). For each cancer type, we fitted a multivariable logistic regression model using the corresponding set of covariates of interest. Based on the logistic regression analyses, odds ratios (ORs) with respect to each predictor and their 95% confidence intervals (CIs) were estimated. After model fitting for each cancer site, we used the Area Under the Receiver Operating Characteristic Curve (AUC) as the primary evaluation metric of the model performance. For each cancer type, we randomly split the full data into training and testing datasets in a 7:3 proportion, and we estimated the AUC, the optimal probability cut point, and several supplementary metrics, including overall accuracy, sensitivity, specificity, positive predictive value (PPV), and negative predictive value (NPV) based on the corresponding optimal cutoff, over 100 random-splitting simulations. Further, we developed nomograms and a Webtool for each cancer type based on the logistic regression models to predict BM at diagnosis as guidance for clinicians. Finally, we also calculated the estimated risk of BM at diagnosis based on logistic regressions for each patient and summarized the characteristic distributions in 3 risk subgroups (<1% [low], 1–10% [intermediate], and >10% [high]) for each cancer type. The cutoffs between low, intermediate, and high risk are arbitrary, as there is no well-defined pretest probability for which brain MRI is recommended, though the authors feel that most clinicians would not pursue a brain MRI if risk is <1%, and most physicians would recommend a brain MRI if risk is >10%. All the analyses were conducted in statistical software R Version 4.1. We used R packages “pROC” (version 1.18.4) for estimating optimal cut points of prediction, and “rms” (version 6.7-0) for assisting in generating the nomograms. *P*-values of less than or equal to 0.05 were regarded as statistically significant.

### Reporting summary

Further information on research design is available in the Nature Portfolio Reporting Summary linked to this article.

## Results

A total of 4,828,305 patients were identified in the NCDB from 2010-2018 (2,095,339 breast cancer, 472,611 melanoma, 627,090 CRC, 407,627 kidney cancer, 164,864 SCLC, and 1,060,774 NSCLC).

**Fig. 1:**
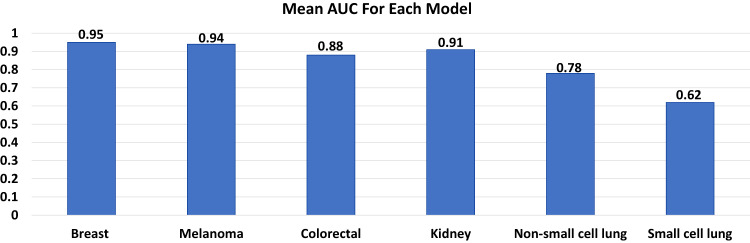
The area under the curve for each model based on 100 random splits of the data. AUC**—**area under the curve.

**Fig. 2:**
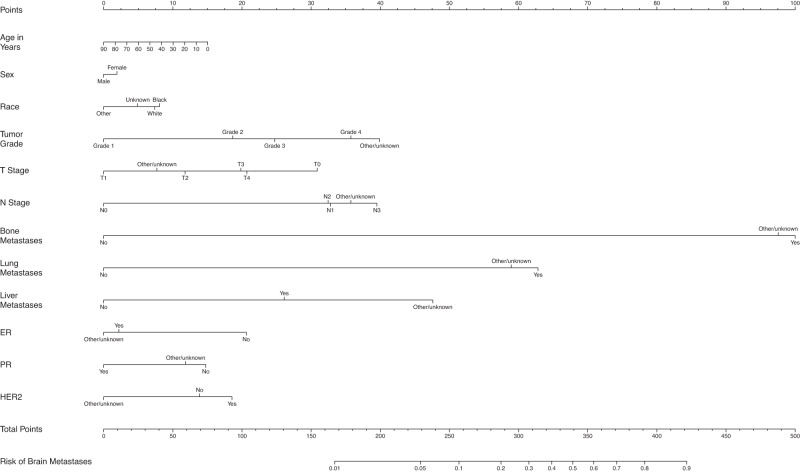
Nomogram for prediction of brain metastases from breast cancer. T stage tumor stage, N Stage nodal stage, ER estrogen receptor, PR progesterone receptor, HER2 human epidermal growth factor receptor 2.

The overall proportion of patients with BM at diagnosis was 0.3%, 1.5%, 0.3%, 1.3%, 16.0%, and 10.3% for breast cancer, melanoma, CRC, kidney cancer, SCLC, and NSCLC, respectively. The incidence of brain-only metastatic disease (without lung, liver, and bone metastasis), was relatively rare for breast cancer (0.06%), melanoma (0.55%), CRC (0.08%), and kidney cancer (0.28%) but did occur more frequently in patients with SCLC (7.78%) and NSCLC (5.10 %). Table 1 shows the demographic and disease-specific data for all patients. The estimates of odds ratios with 95% CI in the LR analyses are shown in Table 2, as well as in the text in the following disease-specific subsections. Supplementary Tables 1 through 6 show the characteristics of patients with each type of cancer stratified by the presence or absence of BM at diagnosis. Figure 1 shows the mean AUC for all models. Figures 2 through 7 show the nomograms developed for different cancer types, and Supplementary Tables 7 through 18 show the nomogram scores associated with each variable level and a reference table for mapping the total scores to predicted BM risks for each cancer type. Supplementary Tables 19 through 24 show the characteristics of cancer-specific populations that are at low (<1%), intermediate (1–10%), and high risk (>10%) of harboring BM at diagnosis. Supplementary Table 25 shows the Mean AUC as well as the 2.5% and 97.5% quantiles for each model based on 100 random splits of the data. Supplementary Table 26 shows model performance metrics for each cancer-specific model. Supplementary Table 27 shows the percentage of patients with brain-only metastatic disease for each of the cancer types. A link to the Webtool for the risk estimation of BM is listed here: https://tinyurl.com/brain-mets.

**Table 1:** Patient Characteristics

	Breast Cancer (*N* = 2,095,339)	Melanoma (*N* = 472,611)	Colon Cancer (*N* = 627,090)	Kidney Cancer (*N* = 407,627)	Non-Small Cell Lung Cancer (*N* = 1,060,774)	Small Cell Lung Cancer (*N* = 164,864)
**Brain metastases at diagnosis**						
No	2088262 (99.7%)	465699 (98.5%)	625084 (99.7%)	402508 (98.7%)	950988 (89.7%)	138505 (84.0%)
Yes	7077 (0.3%)	6912 (1.5%)	2006 (0.3%)	5119 (1.3%)	109786 (10.3%)	26359 (16.0%)
**Patient age**						
Mean (SD)	61.3 (13.0)	63.1 (15.5)	67.3 (14.1)	62.9 (13.8)	68.8 (10.6)	67.5 (9.85)
Median [Min, Max]	62.0 [0, 90.0]	65.0 [0, 90.0]	68.0 [0, 90.0]	64.0 [0, 90.0]	69.0 [0, 90.0]	68.0 [19.0, 90.0]
**Patient sex**						
Male	17829 (0.9%)	275382 (58.3%)	309380 (49.3%)	255114 (62.6%)	541647 (51.1%)	78009 (47.3%)
Female	2077510 (99.1%)	197229 (41.7%)	317710 (50.7%)	152513 (37.4%)	519127 (48.9%)	86855 (52.7%)
**Patient race**						
White	1714434 (81.8%)	460958 (97.5%)	512225 (81.7%)	339784 (83.4%)	899491 (84.8%)	147441 (89.4%)
Black	249881 (11.9%)	2443 (0.5%)	81539 (13.0%)	48484 (11.9%)	116735 (11.0%)	13119 (8.0%)
Other	111696 (5.3%)	4092 (0.9%)	28384 (4.5%)	15600 (3.8%)	37840 (3.6%)	3346 (2.0%)
Unknown	19328 (0.9%)	5118 (1.1%)	4942 (0.8%)	3759 (0.9%)	6708 (0.6%)	958 (0.6%)
**Tumor grade**						
Grade 1	357294 (17.1%)	1162 (0.2%)	60498 (9.6%)	28927 (7.1%)	70542 (6.7%)	252 (0.2%)
Grade 2	735946 (35.1%)	633 (0.1%)	308482 (49.2%)	115569 (28.4%)	197357 (18.6%)	526 (0.3%)
Grade 3	537971 (25.7%)	1342 (0.3%)	83118 (13.3%)	69809 (17.1%)	245370 (23.1%)	13523 (8.2%)
Grade 4	8065 (0.4%)	534 (0.1%)	16002 (2.6%)	25634 (6.3%)	9477 (0.9%)	19460 (11.8%)
Other/unknown	456063 (21.8%)	468940 (99.2%)	158990 (25.4%)	167688 (41.1%)	538028 (50.7%)	131103 (79.5%)
**Clinical T stage**						
T0	9911 (0.5%)	5971 (1.3%)	3658 (0.6%)	777 (0.2%)	4692 (0.4%)	1573 (1.0%)
T1	825262 (39.4%)	127481 (27.0%)	60495 (9.6%)	202831 (49.8%)	290748 (27.4%)	23113 (14.0%)
T2	361479 (17.3%)	49305 (10.4%)	22571 (3.6%)	40606 (10.0%)	236697 (22.3%)	32922 (20.0%)
T3	73295 (3.5%)	27783 (5.9%)	61470 (9.8%)	29276 (7.2%)	130508 (12.3%)	22933 (13.9%)
T4	57510 (2.7%)	19508 (4.1%)	30053 (4.8%)	5633 (1.4%)	150584 (14.2%)	39347 (23.9%)
Other/unknown	767882 (36.6%)	242563 (51.3%)	448843 (71.6%)	128504 (31.5%)	247545 (23.3%)	44976 (27.3%)
**Clinical N stage**						
N0	1468750 (70.1%)	352327 (74.5%)	350310 (55.9%)	291365 (71.5%)	445229 (42.0%)	22649 (13.7%)
N1	198121 (9.5%)	8611 (1.8%)	50496 (8.1%)	17187 (4.2%)	69733 (6.6%)	11847 (7.2%)
N2	32773 (1.6%)	3406 (0.7%)	17090 (2.7%)	1458 (0.4%)	243324 (22.9%)	70431 (42.7%)
N3	23396 (1.1%)	2734 (0.6%)	0 (0%)	94 (0.0%)	97859 (9.2%)	27861 (16.9%)
Other/unknown	372299 (17.8%)	105533 (22.3%)	209194 (33.4%)	97523 (23.9%)	204629 (19.3%)	32076 (19.5%)
**Bone metastases at diagnosis**						
No	2034879 (97.1%)	468483 (99.1%)	619857 (98.8%)	387757 (95.1%)	907643 (85.6%)	129079 (78.3%)
Yes	60006 (2.9%)	3907 (0.8%)	6859 (1.1%)	19586 (4.8%)	150487 (14.2%)	34967 (21.2%)
Other/unknown	454 (0.0%)	221 (0.0%)	374 (0.1%)	284 (0.1%)	2644 (0.2%)	818 (0.5%)
**Lung metastases at diagnosis**						
No	2067340 (98.7%)	463450 (98.1%)	598758 (95.5%)	379222 (93.0%)	940839 (88.7%)	144786 (87.8%)
Yes	26841 (1.3%)	8871 (1.9%)	26960 (4.3%)	27825 (6.8%)	112296 (10.6%)	17843 (10.8%)
Other/unknown	1158 (0.1%)	290 (0.1%)	1372 (0.2%)	580 (0.1%)	7639 (0.7%)	2235 (1.4%)
**Liver metastases at diagnosis**						
No	2072684 (98.9%)	467899 (99.0%)	531661 (84.8%)	397322 (97.5%)	987762 (93.1%)	116882 (70.9%)
Yes	21935 (1.0%)	4491 (1.0%)	94815 (15.1%)	9925 (2.4%)	69450 (6.5%)	47216 (28.6%)
Other/unknown	720 (0.0%)	221 (0.0%)	614 (0.1%)	380 (0.1%)	3562 (0.3%)	766 (0.5%)
**ER (SSF1)**						
Yes	1456995 (69.5%)					
No	296930 (14.2%)					
Other/unknown	341414 (16.3%)					
**PR (SSF2)**						
Yes	1266397 (60.4%)					
No	463133 (22.1%)					
Other/unknown	365809 (17.5%)					
**HER2 (SSF15)**						
Yes	216932 (10.4%)					
No	1201940 (57.4%)					
Other/unknown	676467 (32.3%)					
**Ulceration (SSF2)**						
Yes		52677 (11.1%)				
No		319656 (67.6%)				
Other/unknown		100278 (21.2%)				
**Tumor Histology** ^**a**^						
Type 1			580274 (92.5%)			
Type 2			46816 (7.5%)			
**CEA (SSF1)**						
Positive			152173 (24.3%)			
Negative			161662 (25.8%)			
Other/unknown			313255 (50.0%)			
**Tumor Histology** ^**b**^						
Type 1				71623 (17.6%)		
Type 2				288011 (70.7%)		
Type 3				47993 (11.8%)		
**Sarcomatoid Features (SSF4)**						
Yes				11391 (2.8%)		
No				257570 (63.2%)		
Other/unknown				138666 (34.0%)		
**Fuhrman Nuclear Grade (SSF6)**						
1				28171 (6.9%)		
2				125887 (30.9%)		
3				69877 (17.1%)		
4				19545 (4.8%)		
Other/unknown				164147 (40.3%)		
**Tumor Histology** ^**c**^						
Adenocarcinoma					530344 (50.0%)	
Squamous Cell Carcinoma					297015 (28.0%)	
Other/unknown					233415 (22.0%)	

^a^For colon histology,

Type 1 = Adenocarcinoma + carcinoma + tubulovillous adenocarcinoma + mucinous adenocarcinoma

Type 2 = Neuroendocrine + Other/unknown

^b^For RCC histology,

Type 1 = Adenocarcinoma + papillary adenocarcinoma.

Type 2 = Renal cell carcinoma.

Type 3 = Urothelial cell carcinoma + Other/unknown.

^c^Tumor histology for non-small cell lung cancer.

**Table 2:** Odds Ratios for Prediction of Brain Metastases at Diagnosis

	OR (95% CI)					
Cancer Site	Breast Cancer	Melanoma	Colorectal Cancer	Kidney Cancer	Non-Small Cell Lung Cancer	Small Cell Lung Cancer
**Patient age**						
1-year increase	**0.996** (0.994–0.997)	**0.987** (0.985–0.989)	**0.993** (0.990–0.996)	**0.992** (0.990–0.994)	**0.968** (0.967–0.968)	**0.974** (0.973–0.976)
**Patient sex (ref** **=** **Male)**						
Female	1.053 (0.832–1.331)	**0.661** (0.621–0.703)	0.985 (0.900–1.078)	**0.875** (0.821–0.932)	**1.013 (1.000–1.027)**	**0.895** (0.871–0.919)
**Patient race (ref** **=** **White)**						
Black	1.020 (0.954–1.090)	**0.550** (0.388–0.779)	**0.734** (0.641–0.841)	**0.693** (0.621–0.773)	1.001 (0.981–1.022)	**1.112** (1.060–1.167)
Other	**0.821** (0.726–0.929)	0.999 (0.757–1.320)	0.798 (0.636–1.002)	1.036 (0.897–1.197)	**1.156** (1.119–1.193)	1.008 (0.917–1.108)
Unknown	0.937 (0.715–1.227)	0.904 (0.671–1.216)	0.756 (0.441–1.296)	1.063 (0.791–1.428)	**1.124** (1.039–1.216)	**1.204** (1.02–1.422)
**Tumor grade (ref** **=** **Grade 1)**						
Grade 2	**1.645** (1.416–1.911)	1.597 (0.372–6.844)	1.184 (0.903–1.552)	1.186 (0.823–1.709)	**2.118** (1.999–2.244)	0.853 (0.546–1.334)
Grade 3	**1.934** (1.663–2.249)	**2.638** (1.061–6.561)	**2.702** (2.050–3.562)	**1.528** (1.079–2.164)	**3.929** (3.718–4.151)	0.958 (0.668–1.375)
Grade 4	**2.596** (1.790–3.766)	1.069 (0.351–3.259)	**2.715** (1.884–3.914)	1.199 (0.836–1.720)	**4.011** (3.686–-4.365)	0.897 (0.625–1.286)
Other/unknown	**2.900** (2.485–3.384)	1.186 (0.495–2.839)	**2.933** (2.249–3.824)	**1.672** (1.203–2.325)	**4.433** (4.198–4.68)	1.056 (0.738–1.511)
**Clinical T stage (ref** = **T1)**						
T0	**2.280** (1.846–2.817)	**19.342** (15.759–23.739)	**3.254** (2.260–4.685)	**2.368** (1.617–3.47)	**2.096** (1.932–2.274)	**1.265** (1.095–1.461)
T2	**1.369** (1.242–1.509)	**1.506** (1.142–1.987)	0.798 (0.509–1.252)	**2.384** (2.162–2.629)	**1.823** (1.781–1.865)	**1.290** (1.226–1.358)
T3	**1.698** (1.515–1.904)	**2.327** (1.784–3.036)	1.036 (0.799–1.344)	**1.699** (1.524–1.893)	**1.738** (1.694–1.783)	**1.227** (1.162–1.297)
T4	**1.738** (1.567–1.927)	**4.897** (3.878–6.184)	1.009 (0.773–1.317)	**1.802** (1.562–2.079)	**1.724** (1.682–1.768)	**1.276** (1.214–1.341)
Other/unknown	**1.228** (1.101–1.370)	**4.197** (3.466–5.083)	1.179 (0.956–1.455)	**1.210** (1.085–1.350)	**1.439** (1.398–1.481)	**1.254** (1.185–1.328)
**Clinical N stage (ref** = **N0)**						
N1	**2.399** (2.214–2.599)	**1.557** (1.362–1.779)	**1.505** (1.304–1.738)	**1.159 (1.064–1.263)**	**1.934** (1.880–1.989)	1.049 (0.984–1.118)
N2	**2.377** (2.123–2.662)	**1.588** (1.291–1.954)	**1.989** (1.635–2.418)	0.726 (0.474–1.113)	**2.124** (2.083–2.166)	0.993 (0.949–1.038)
N3	**2.870** (2.575–3.199)	**1.999** (1.674–2.387)	**-**	0.328 (0.044–2.434)	**2.036** (1.989–2.085)	0.953 (0.906–1.004)
Other/unknown	**2.597** (2.359–2.858)	**1.443** (1.34–1.554)	**1.227** (1.094–1.376)	1.070 (0.972–1.178)	**1.671** (1.625–1.719)	**1.214** (1.144–1.288)
**Bone metastases at diagnosis (ref** = **No)**						
Yes	**14.422** (13.475–15.435)	**2.009** (1.815–2.223)	**5.381** (4.769–6.070)	**2.231** (2.084–2.389)	**1.906** (1.876–1.936)	**1.435** (1.389–1.482)
Other/unknown	**13.510** (10.054–18.154)	**3.603** (2.387–5.438)	**7.061** (4.756–10.483)	**3.390** (2.435–4.720)	**3.116** (2.846–3.411)	**2.157** (1.840–2.529)
**Lung metastases at diagnosis (ref** = **No)**						
Yes	**5.345** (5.021–5.690)	**23.399** (21.858–25.049)	**9.763** (8.700–10.957)	**10.481** (9.744–11.273)	**1.530** (1.502–1.558)	**1.650** (1.586–1.716)
Other/unknown	**4.821** (3.993–5.821)	**22.625** (16.548–30.934)	**4.465** (3.060–6.514)	**12.758** (10.002–16.274)	**2.222** (2.104–2.347)	**2.125** (1.930–2.340)
**Liver metastases at diagnosis (ref** = **No)**						
Yes	**2.007** (1.882–2.141)	**1.677** (1.523–1.848)	**1.411** (1.257–1.583)	**1.256** (1.154–1.366)	**1.773** (1.737–1.810)	1.005 (0.975–1.037)
Other/unknown	**3.562** (2.886–4.396)	**4.321** (2.868–6.510)	**5.298** (3.498–8.026)	**2.633** (2.014–3.441)	**3.176** (2.939–3.432)	**2.535** (2.160–2.974)
**ER (SSF1) (ref** = **No)**						
Yes	**0.612** (0.563–0.664)					
Other/unknown	**0.577** (0.440–0.757)					
**PR (SSF2) (ref** = **No)**						
Yes	**0.674** (0.624–0.729)					
Other/unknown	0.925 (0.712–1.202)					
**HER2 (SSF15) (ref** = **No)**						
Yes	**1.133** (1.060–1.211)					
Other/unknown	**0.691** (0.619–0.771)					
**Ulceration (SSF2) (ref** = **No)**						
Yes		**2.193** (1.923–2.502)				
Other/unknown		**4.998** (4.580–5.455)				
**CEA (SSF1) (ref** **=** **Negative)**						
Positive			**1.829** (1.539–2.173)			
Other/unknown			**1.425** (1.200–1.691)			
**Tumor histology** ^a^ **(ref** **=** **Type 1)**						
Type 2			0.983 (0.817–1.182)			
**Sarcomatoid Features (SSF4) (ref** = **No)**						
Yes				**1.355** (1.179–1.557)		
Other/unknown				**1.835** (1.661–2.027)		
**Fuhrman Nuclear Grade (SSF6) (ref** = 1)						
2				0.872 (0.606–1.256)		
3				1.090 (0.765–1.553)		
4				**1.469** (1.020–2.115)		
Other/unknown				**1.581** (1.129–2.215)		
**Tumor histology** ^b^ **(ref** **=** **Type 1)**						
Type 2				**2.535** (2.214–2.901)		
Type 3				1.099 (0.933–1.296)		
**Tumor histology** ^**c**^ **(ref=** **Adenocarcinoma)**						
Squamous Cell Carcinoma					**0.756** (0.743–0.768)	
Other/unknown					**0.363** (0.356–0.370)	

Bolded odds ratios signify statistical significance.

^a^For colon cancer histology,

Type 1 = Adenocarcinoma + carcinoma + tubulovillous adenocarcinoma + mucinous adenocarcinoma.

Type 2 = Neuroendocrine + Other/unknown.

^b^For kidney cancer histology,

Type 1 = Adenocarcinoma + papillary adenocarcinoma.

Type 2 = Renal cell carcinoma.

Type 3 = Urothelial cell carcinoma + Other/unknown.

^c^Tumor histology for non-small cell lung cancer.

**Fig. 3:**
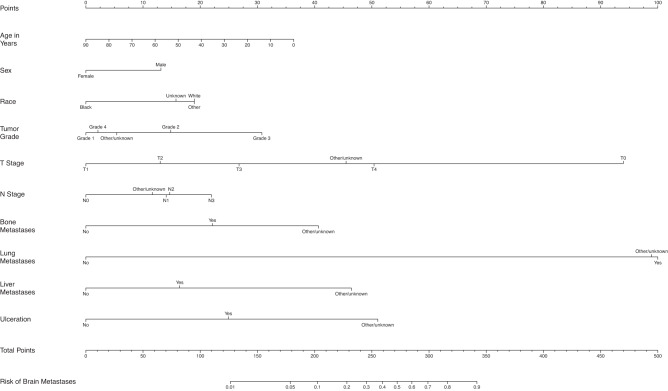
Nomogram for prediction of brain metastases from melanoma. T stage tumor stage, N Stage nodal stage.

**Fig. 4:**
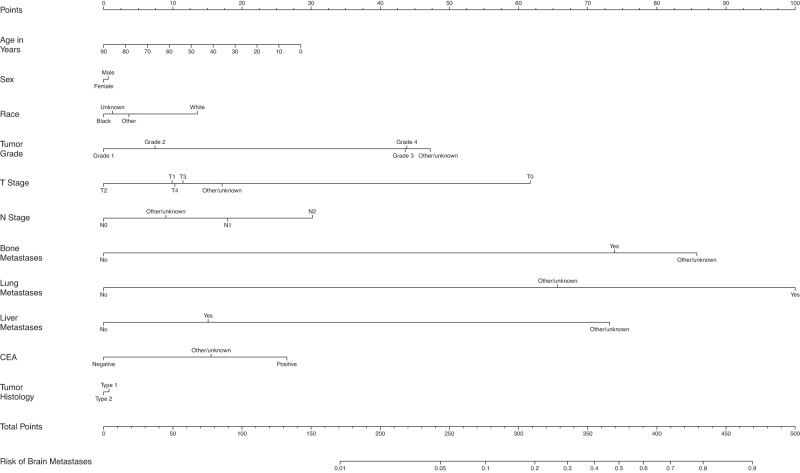
Nomogram for prediction of brain metastases from colorectal cancer. T stage tumor stage; N Stage nodal stage. CEA carcinoembryonic antigen.

**Fig. 5:**
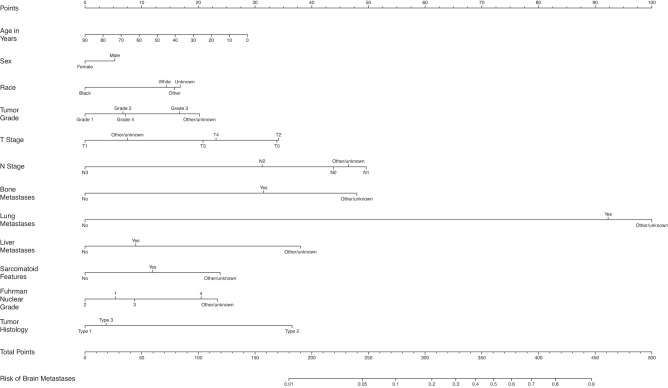
Nomogram for prediction of brain metastases from kidney cancer. T stage *tumor stage*; N Stage nodal stage.

**Fig. 6:**
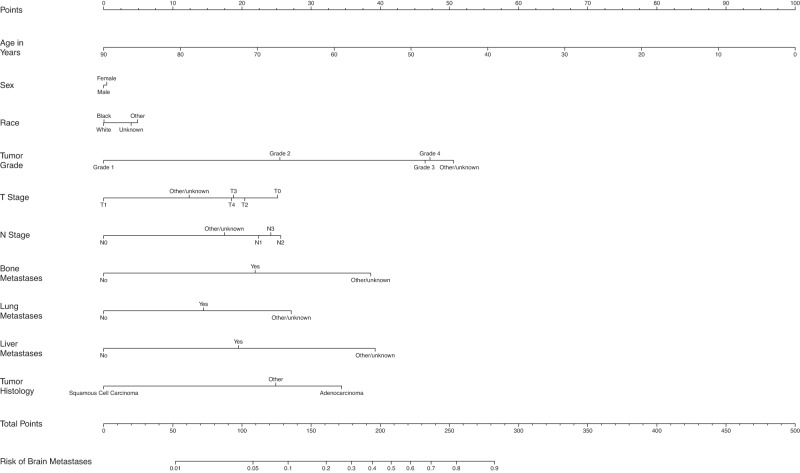
Nomogram for prediction of brain metastases from non-small cell lung cancer. T stage tumor stage, N Stage nodal stage.

**Fig. 7:**
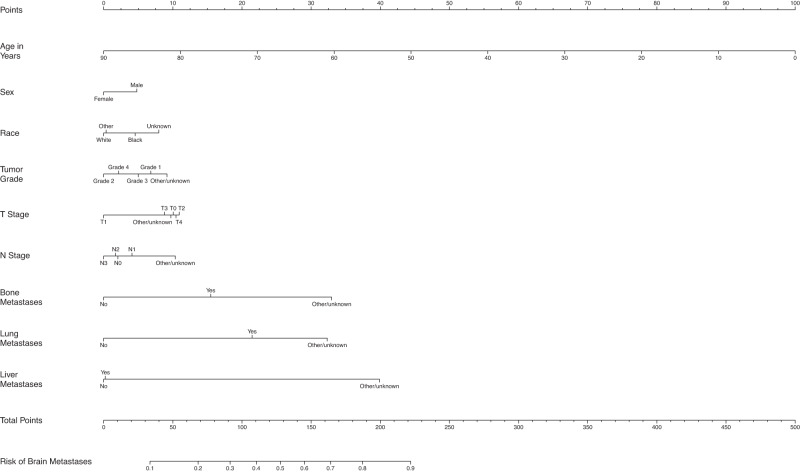
Nomogram for prediction of brain metastases from small cell lung cancer. T stage tumor stage, N Stage nodal stage.

### Breast cancer

For patients diagnosed with breast cancer, those with BM were more likely to be black race (18.3% vs. 11.9%), have high grade tumors (G3/4 35.4% vs. 26.0%) and more advanced T (T3/4 36.8% vs. 6.2%) and N stage (N2/3 20.0% vs. 2.6%), as well as metastases to bone (65.0% vs. 2.7%), lung (44.4% vs. 1.1%), and liver at diagnosis (31.1% vs. 0.9%). Patients with BM were also more likely to have estrogen receptor (ER) negative (30.0% vs. 14.1%), progesterone receptor (PR) negative (40.9% vs. 22.0%), and human epidermal growth factor receptor 2 (HER2) positive disease (22.5% vs. 10.3%).

Those with tumor grade 2, 3, and 4 respectively had 1.65 (95% CI: 1.42–1.91), 1.93 (95% CI: 1.66–2.25), 2.60 (95% CI: 1.79–3.77) times the odds for BM compared to those with tumor grade 1. Having T1 disease was associated with lower odds of having BM compared to having clinical T0 or T2-4 disease, while patients with N0 disease had lower odds of having BM compared to those with N1-N3 disease. Those with bone metastases had 14.42 (95% CI: 13.48–15.44) times the odds for BM compared to those without bone metastases. Those with lung metastases had 5.35 (95% CI: 5.02–5.69) times the odds for BM compared to those without lung metastases. Those with liver metastases had 2.01 (95% CI: 1.88–2.14) times the odds for BM compared to those without. Patients with ER+ disease had lower odds of BM with OR of 0.61 (95% CI: 0.56–0.66). Similarly, those with PR+ disease had lower odds of BM with a OR of 0.67 (95% CI: 0.62–0.73). HER2+ was associated with a slightly higher odds to have BM (OR = 1.13, 95% CI: 1.06–1.21) compared to HER2- disease. Across the 100 7:3 training/testing random splits, the model showed an average AUC of 0.95 (Fig. 1).

### Melanoma

For patients diagnosed with melanoma, those with BM were more likely to be male (71.3% vs. 58.1%), to have high-grade tumors (G3/4 1.8% vs. 0.4%), and to have clinical T stage 0 (22.2% vs. 1.0%). Additionally, they were more likely to have more advanced nodal disease (N2/3 5.7% vs. 1.2%), metastatic disease to bone (19.1% vs. 0.6%), lung (52.5% vs. 1.1%), and liver (21.4% vs. 0.6%) at diagnosis (Figs. 3–7).

Female patients had 0.66 (95% CI: 0.62–0.70) times the odds to have BM compared to males. Those with grade 3 tumors had about 2.64 (95% CI: 1.06–6.56) times the odds for BM compared to those with grade 1. Patients with T1 disease had lower odds of having BM compared to T0, T3, and T4 disease and those with N0 disease had lower odds of having BM compared to those with N1-3 disease. Furthermore, those with bone metastases had 2.01 (95% CI: 1.82–2.22) times the odds for BM compared to those without bone metastases. Those with lung metastases had 23.40 (95% CI: 21.86–25.05) times the odds for BM compared to those without lung metastases. And those with liver metastases had 1.68 (95% CI: 1.52–1.85) times the odds for BM compared to those without. Patients having tumor ulceration had 2.19 (95% CI: 1.92–2.50) times the odds to get BM compared to those without ulceration. The average AUC across 100 random splits is 0.94 (Fig. 1).

### Colorectal cancer

For patients diagnosed with CRC, those with BM were more likely to be younger (65.4 vs. 67.3) and male (52.5% vs. 49.3%). They were more likely to have higher grade tumors (G3/4 20.6% vs. 15.8%) and with higher T (T4 7.1% vs. 4.8%) and N stage (N2 7.7% vs. 2.7%). Additionally, they were more likely to have bone (22.8%, vs. 1.0%), lung (50.9% vs. 4.1%), and liver metastases at diagnosis (54.1% vs. 15.0%), with positive CEA (45.7% vs. 24.2%).

Black race conferred 0.73 (95% CI: 0.64–0.84) times the odds to have BM compared to white race. Those with grade 3 and 4 tumors had 2.70 (95% CI: 2.05–3.56) and 2.72 (95% CI: 1.88–3.91) times the odds of BM compared to those with grade 1 tumors. Patients with T0 disease had 3.25 times the odds of having BM compared to those with T1 disease (95% CI: 2.26–4.69), while those with N0 disease had lower odds of BM compared to those with N1-2 disease. In addition, those with bone metastases had 5.38 (95% CI: 4.77–6.07) times the odds of BM compared to those without bone metastases. Those with lung metastases had 9.76 (95% CI: 8.70–10.96) times the odds for BM compared to those without lung metastases. And those with liver metastases had 1.41 (95% CI: 1.26–1.58) times the odds of BM compared to those without liver metastases. Patients with positive CEA had 1.83 (95% CI: 1.54–2.17) times the odds to get BM compared to normal CEA. The average AUC across 100 random splits is 0.89 (Fig. 1).

### Kidney cancer

For patients with kidney cancer, those with BM were more likely to be male (69.0% vs. 62.5%) and white race (87.0% vs. 83.3%). They also were more likely to have higher N stage (N1-3 21.1% vs. 4.4%) as well as have bone (36.2% vs. 4.4%), lung (63.8% vs. 6.1%), and liver metastases (17.8% vs. 2.2%) at diagnosis. A higher proportion of patients with BM had sarcomatoid features (7.2% vs. 2.7%) and grade 4 Fuhrman nuclear grade (8.6% vs. 4.7%). Histology for patients with BM was more likely to be renal cell carcinoma (RCC) as compared to adenocarcinoma/papillary adenocarcinoma or urothelial carcinoma / other (86.7% vs. 70.5%).

Female patients had slightly lower odds of BM compared to males (OR = 0.88, 95% CI: 0.82–0.93), and black patients had lower odds compared to white patients (OR = 0.69, 95% CI: 0.62–0.77). Those with tumor grade 3 tumors had 1.53 (95% CI: 1.08–2.16) times the odds for BM compared to those with grade 1 tumors. Patients with T1 disease were associated with lower odds of having BM compared to those having T0 or T2-4 disease, while those with N0 disease had lower odds of having BM compared to those with N1 disease. Those with bone metastases had about double the odds of BM (OR = 2.23, 95% CI: 2.08–2.39) compared to those without bone metastases. Those with lung metastases had 10.48 (95% CI: 9.74–11.27) times the odds for BM compared to those without lung metastases. Also, those with liver metastases had 1.26 (95% CI: 1.15–1.37) times the odds for BM compared to those without liver metastases. Patients with sarcomatoid features had 1.36 (95% CI: 1.18–1.56) times the odds of getting BM compared to those without sarcomatoid features. Having RCC was associated with 2.54 times the odds to have BM (95% CI: 2.21–2.90) compared to having adenocarcinoma or papillary adenocarcinoma histology. The average AUC across 100 random splits is 0.91 (Fig. 1).

### Non-small cell lung cancer

For patients with NSCLC, those with BM were more likely to be younger (64.9 vs. 69.3) and black race (12.7% vs. 10.8%). BM were more common in patients with unknown grade (66.2% vs. 48.9%), as well as more advanced T (T3/4 36.5% vs. 25.3%) and N stage (N2/3 51.5% vs. 29.9%), and those with bone (33.5% vs. 12.0%), lung (21.6% vs. 9.3%), and liver metastases (16.9% vs. 5.3%) at diagnosis. Patients diagnosed with BM also had a higher proportion of adenocarcinoma histology compared to those without BM (65.4% vs. 48.2%).

Female patients had slightly higher odds of BM compared to males (OR = 1.03, 95% CI: 1.00–1.03). Patients with grade 2 to 4 tumors, respectively, had 2.12 (95% CI: 2.00–2.24), 3.93 (95% CI: 3.72–4.15), and 4.01 (95% CI: 3.69–4.37) times the odds for BM compared to those with grade 1 tumors. Having T1 disease was associated with lower odds of having BM compared to having T0 or T2-4 disease, while having N0 disease had lower odds of having BM compared to having N1-3 disease. Those with bone metastases had 1.91 (95% CI: 1.88–1.94) times the odds of BM compared to those without bone metastases. Those with lung metastases had 1.53 (95% CI: 1.50–1.56) times the odds for BM compared to those without lung metastases. Also, those with liver metastases had 1.77 (95% CI: 1.74–1.81) times the odds for BM compared to those without liver metastases. Patients with squamous cell carcinoma had about 0.76 times the odds to get BM compared to those having adenocarcinoma histology (95% CI: 0.74–0.77). The average AUC across 100 random splits is 0.78 (Fig. 1).

### Small cell lung cancer

For patients with SCLC, those with BM were more likely to be younger (65.4 vs. 67.9), males (50.3% vs. 46.8%), and black race (8.8% vs. 7.8%). Patients with BM were also more likely to have an unknown grade (82.2% vs. 79.0%), higher T stage (T4, 25.5% vs. 23.5%), and unknown N stage (23.1% vs. 18.8%). Additionally, patients with BM were more likely to have bone (28.2% vs. 19.9%), liver (32.1% vs. 28.0%), and lung metastases (15.9% vs. 9.9%) at diagnosis.

Female patients had slightly lower odds of BM compared to males (OR = 0.90, 95% CI: 0.87–0.92) and black patients had slightly higher odds compared to white patients (OR = 1.11, 95% CI: 1.06–1.17). Patients with T1 disease had lower odds of BM compared to those with T0 or T2-4 disease, and those with N0 disease had higher odds of having BM compared to those with N3 disease. Those with bone metastases had 1.44 (95% CI: 1.39–1.48) times the odds of BM compared to those without bone metastases. Those with lung metastases had 1.65 (95% CI: 1.59–1.72) times the odds for BM compared to those without lung metastases. In addition, those with liver metastases did not have significantly higher odds for BM compared to those without liver metastases. The average AUC across 100 random splits is 0.62 (Fig. 1).

## Discussion

Limited resources exist to estimate the risk of BM at the time of initial cancer diagnosis, and only SCLC and NSCLC have clear recommendations in the NCCN regarding the use of brain MRI for staging. In this work, we comprehensively studied of the presence of brain metastases in multiple cancer types based on clinical and pathologic factors. This study successfully developed and validated disease-specific models to predict the presence of BM in patients with a new cancer diagnosis. The models for breast cancer, melanoma, kidney cancer, and CRC exhibited excellent to outstanding discrimination^25^ with average AUC values based on random training/testing data splitting all larger than 0.87. The models for SCLC had poor discrimination (average AUC at 0.62), and the model for NSCLC showed acceptable discrimination (average AUC at 0.78). This study can be incorporated into guidelines for cancer staging and the nomograms and webtools developed based on our models will aid oncologists in the clinic by giving a pre-test probability of the presence of BM when considering brain imaging.

Detailing the multiplicity of cancer type-specific clinical and histological variables that confer a high (>10%) risk of harboring BM in current staging guidelines would be cumbersome. The generated nomograms and associated web application assist with the operationalization of our findings and will aid with the clinical decision to obtain a brain MRI as part of initial staging work-up. In addition, Supplementary Tables 13 through 18 show the characteristics of patients with each type of cancer who have either <1%, 1–10%, or >10% estimated risk of having BM. As expected, populations with a >10% risk of BM generally have a higher proportion of patients with bone, liver, and lung metastases as well as more advanced T and N stage.

The model developed for SCLC warrants further discussion given its poor discrimination with the average AUC as low as 0.6. The authors feel this is representative of the biology of SCLC, as it is known that SCLC has a high propensity for brain metastasis^26^. This is reflected in Supplementary Table 24, which shows that there are no individuals in our study that had a <1% risk of having BM as predicted by the nomogram. This supports the NCCN recommendation of screening brain MRI for all patients diagnosed with SCLC, since the likelihood of brain metastases is relatively high and a highly accurate nomogram could not be generated to discriminate between the presence and absence of brain metastasis at diagnosis.

The other models developed herein compare favorably to prior models predicting BM. A nomogram to predict BM from newly diagnosed breast cancer utilizing the surveillance, epidemiology, and end results (SEER) database demonstrated an AUC of 0.64^27^ as compared to our AUC of about 0.95 for breast cancer. Zhang et al used the SEER database to develop and validate a nomogram for squamous cell carcinoma of the lung with an AUC of 0.8^28^, paralleling our NSCLC model AUC of about 0.78. There are limited data to predict for BM at diagnosis beyond these reports, underscoring the utility of our work.

This study has several limitations. First, the NCDB does not capture CNS symptoms at diagnosis. Certainly, patients with symptomatic disease in the brain are more likely to receive a brain MRI and be subsequently diagnosed and coded as having BM. Thus, the models and nomograms generated may overestimate the risk of BM in patients that are asymptomatic, particularly for cancers other than SCLC and NSCLC where brain MRI screening is recommended in the NCCN guidelines. However, conversely, since MRI screening is not utilized across all patients, it is possible that the model may underestimate the true rate of brain metastases as some patients may have harbored asymptomatic brain metastases but did not have MRI screening. Additionally, we included patients with missing data in this study. As seen in the nomograms, multiple variables include “unknown” as a category, and in general the unknown category is more likely to be associated with BM. The authors propose that the reasoning for this may be reflective of clinical practice when a patient is diagnosed with BM. For example, if a patient presents with BM, the primary tumor characteristics such as grade, T stage, and N stage no longer play a strong role in treatment recommendation, and as such may not be documented or coded appropriately and thus listed as “unknown.” Also, the NCDB does not contain information regarding driver mutations which may affect biologic aggressiveness and risk of brain metastasis ^29,30^. And, some variables within the models may be inherently correlated (ex. Triple negative breast cancer, high grade, and black race), potentially resulting in some variables not being associated with brain metastases. We elected to keep all baseline demographic and tumor variables in the models regardless of their association with brain metastases for coherency of the models across cancer types. Lastly, most patients with brain metastasis in our study also had metastasis to liver, lung, and/or bone. As such, predictive power may be less in patients without evidence of other metastatic disease, particularly in kidney, breast, colorectal cancer, and melanoma, where brain-only metastatic disease is less common.

In conclusion, we developed and validated models that predict the presence of BM at diagnosis for patients diagnosed with breast cancer, melanoma, CRC, kidney cancer, NSCLC and SCLC. This work can be referred to in guidelines for cancer staging and the nomograms and Webtools can guide clinicians in the decision to obtain brain MRI as a part of their staging work-up.

### Supplementary information

Supplementary Information

Supplementary Data 1

Description of Additional Supplementary Files

Reporting Summary

## Data Availability

The data analyzed during this study are publicly available via request of the NCDB Participant Use File (PUF) from the Commission on Cancer (CoC). Herein we utilized 2021 NCDB PUFs for breast cancer, melanoma, kidney cancer, CRC, SCLC, and NSCLC and included patients diagnosed from 2010-2018. The source data for the figures are available as Supplementary Data 1. All other data are available from the corresponding author on reasonable request.
