# Supplementary material for: Estimating the risk of brain metastasis for patients newly diagnosed with cancer
Source: Commun Med (Lond). 2024 Feb 22;4:27. doi: 10.1038/s43856-024-00445-7 (PMC10883934; doi:10.1038/s43856-024-00445-7)
Supplement: Supplementary file 1 — Supplementary Information [file 43856_2024_445_MOESM1_ESM.docx]

**Supplementary Table 1 - Breast Data Stratified by Presence or Absence of Brain Metastasis at Diagnosis**

|  | **Absence of brain metastases**  **(N=2088262)** | **Presence of brain metastases**  **(N=7077)** | **P-value** |
| --- | --- | --- | --- |
| **Patient age** |  |  | **<0.001** |
| Mean (SD) | 61.3 (13.0) | 60.6 (12.7) |  |
| Median [Min, Max] | 62.0 [0, 90.0] | 61.0 [21.0, 90.0] |  |
| **Patient sex** |  |  | **0.012** |
| Male | 17749 (0.9%) | 80 (1.1%) |  |
| Female | 2070513 (99.2%) | 6997 (98.9%) |  |
| **Patient race** |  |  | **<0.001** |
| White | 1709017 (81.8%) | 5417 (76.5%) |  |
| Black | 248586 (11.9%) | 1295 (18.3%) |  |
| Other | 111393 (5.3%) | 303 (4.3%) |  |
| Unknown | 19266 (0.9%) | 62 (0.9%) |  |
| **Tumor grade** |  |  | **<0.001** |
| Grade 1 | 357088 (17.1%) | 206 (2.9%) |  |
| Grade 2 | 734400 (35.2%) | 1546 (21.8%) |  |
| Grade 3 | 535511 (25.6%) | 2460 (34.8%) |  |
| Grade 4 | 8025 (0.4%) | 40 (0.6%) |  |
| Other/unknown | 453238 (21.7%) | 2825 (39.9%) |  |
| **Clinical T stage** |  |  | **<0.001** |
| T0 | 9785 (0.5%) | 126 (1.8%) |  |
| T1 | 824524 (39.5%) | 738 (10.4%) |  |
| T2 | 360144 (17.2%) | 1335 (18.9%) |  |
| T3 | 72516 (3.5%) | 779 (11.0%) |  |
| T4 | 55684 (2.7%) | 1826 (25.8%) |  |
| Other/unknown | 765609 (36.7%) | 2273 (32.1%) |  |
| **Clinical N stage** |  |  | **<0.001** |
| N0 | 1467421 (70.3%) | 1329 (18.8%) |  |
| N1 | 195835 (9.4%) | 2286 (32.3%) |  |
| N2 | 32141 (1.5%) | 632 (8.9%) |  |
| N3 | 22609 (1.1%) | 787 (11.1%) |  |
| Other/unknown | 370256 (17.7%) | 2043 (28.9%) |  |
| **Bone metastases at diagnosis** |  |  | **<0.001** |
| No | 2032474 (97.3%) | 2405 (34.0%) |  |
| Yes | 55406 (2.7%) | 4600 (65.0%) |  |
| Other/unknown | 382 (0.0%) | 72 (1.0%) |  |
| **Lung metastases at diagnosis** |  |  | **<0.001** |
| No | 2063597 (98.8%) | 3743 (52.9%) |  |
| Yes | 23699 (1.1%) | 3142 (44.4%) |  |
| Other/unknown | 966 (0.0%) | 192 (2.7%) |  |
| **Liver metastases at diagnosis** |  |  | **<0.001** |
| No | 2067981 (99.0%) | 4703 (66.5%) |  |
| Yes | 19735 (0.9%) | 2200 (31.1%) |  |
| Other/unknown | 546 (0.0%) | 174 (2.5%) |  |
| **ER (SSF1)** |  |  | **<0.001** |
| Yes | 1453511 (69.6%) | 3484 (49.2%) |  |
| No | 294804 (14.1%) | 2126 (30.0%) |  |
| Other/unknown | 339947 (16.3%) | 1467 (20.7%) |  |
| **PR (SSF2)** |  |  | **<0.001** |
| Yes | 1263743 (60.5%) | 2654 (37.5%) |  |
| No | 460240 (22.0%) | 2893 (40.9%) |  |
| Other/unknown | 364279 (17.4%) | 1530 (21.6%) |  |
| **HER2 (SSF15)** |  |  | **<0.001** |
| Yes | 215337 (10.3%) | 1595 (22.5%) |  |
| No | 1198310 (57.4%) | 3630 (51.3%) |  |
| Other/unknown | 674615 (32.3%) | 1852 (26.2%) |  |

**Supplementary Table 2 - Melanoma Data Stratified by Presence or Absence of Brain Metastasis at Diagnosis**

|  | **Absence of brain metastases**  **(N=465699)** | **Presence of brain metastases**  **(N=6912)** | **P-value** |
| --- | --- | --- | --- |
| **Patient age** |  |  | 0.330 |
| Mean (SD) | 63.0 (15.5) | 63.2 (14.1) |  |
| Median [Min, Max] | 65.0 [0, 90.0] | 64.0 [0, 90.0] |  |
| **Patient sex** |  |  | **<0.001** |
| Male | 270455 (58.1%) | 4927 (71.3%) |  |
| Female | 195244 (41.9%) | 1985 (28.7%) |  |
| **Patient race** |  |  | **0.012** |
| White | 454223 (97.5%) | 6735 (97.4%) |  |
| Black | 2396 (0.5%) | 47 (0.7%) |  |
| Other | 4019 (0.9%) | 73 (1.1%) |  |
| Unknown | 5061 (1.1%) | 57 (0.8%) |  |
| **Tumor grade** |  |  | **<0.001** |
| Grade 1 | 1156 (0.2%) | 6 (0.1%) |  |
| Grade 2 | 630 (0.1%) | 3 (0.0%) |  |
| Grade 3 | 1231 (0.3%) | 111 (1.6%) |  |
| Grade 4 | 521 (0.1%) | 13 (0.2%) |  |
| Other/unknown | 462161 (99.2%) | 6779 (98.1%) |  |
| **Clinical T stage** |  |  | **<0.001** |
| T0 | 4439 (1.0%) | 1532 (22.2%) |  |
| T1 | 127359 (27.3%) | 122 (1.8%) |  |
| T2 | 49215 (10.6%) | 90 (1.3%) |  |
| T3 | 27664 (5.9%) | 119 (1.7%) |  |
| T4 | 19178 (4.1%) | 330 (4.8%) |  |
| Other/unknown | 237844 (51.1%) | 4719 (68.3%) |  |
| **Clinical N stage** |  |  | **<0.001** |
| N0 | 350283 (75.2%) | 2044 (29.6%) |  |
| N1 | 8172 (1.8%) | 439 (6.4%) |  |
| N2 | 3256 (0.7%) | 150 (2.2%) |  |
| N3 | 2493 (0.5%) | 241 (3.5%) |  |
| Other/unknown | 101495 (21.8%) | 4038 (58.4%) |  |
| **Bone metastases at diagnosis** |  |  | **<0.001** |
| No | 463044 (99.4%) | 5439 (78.7%) |  |
| Yes | 2589 (0.6%) | 1318 (19.1%) |  |
| Other/unknown | 66 (0.0%) | 155 (2.2%) |  |
| **Lung metastases at diagnosis** |  |  | **<0.001** |
| No | 460332 (98.8%) | 3118 (45.1%) |  |
| Yes | 5239 (1.1%) | 3632 (52.5%) |  |
| Other/unknown | 128 (0.0%) | 162 (2.3%) |  |
| **Liver metastases at diagnosis** |  |  | **<0.001** |
| No | 462630 (99.3%) | 5269 (76.2%) |  |
| Yes | 3012 (0.6%) | 1479 (21.4%) |  |
| Other/unknown | 57 (0.0%) | 164 (2.4%) |  |
| **Ulceration (SSF2)** |  |  | **<0.001** |
| Yes | 52142 (11.2%) | 535 (7.7%) |  |
| No | 318725 (68.4%) | 931 (13.5%) |  |
| Other/unknown | 94832 (20.4%) | 5446 (78.8%) |  |

**Supplementary Table 3 - Colorectal Data Stratified by Presence or Absence of Brain Metastasis at Diagnosis**

|  | **Absence of brain metastases**  **(N=625084)** | **Presence of brain metastases**  **(N=2006)** | **P-value** |
| --- | --- | --- | --- |
| **Patient age** |  |  | **<0.001** |
| Mean (SD) | 67.3 (14.1) | 65.4 (12.8) |  |
| Median [Min, Max] | 68.0 [0, 90.0] | 65.0 [4.00, 90.0] |  |
| **Patient sex** |  |  | **0.005** |
| Male | 308327 (49.3%) | 1053 (52.5%) |  |
| Female | 316757 (50.7%) | 953 (47.5%) |  |
| **Patient race** |  |  | 0.729 |
| White | 510571 (81.7%) | 1654 (82.5%) |  |
| Black | 81283 (13.0%) | 256 (12.8%) |  |
| Other | 28302 (4.5%) | 82 (4.1%) |  |
| Unknown | 4928 (0.8%) | 14 (0.7%) |  |
| **Tumor grade** |  |  | **<0.001** |
| Grade 1 | 60437 (9.7%) | 61 (3.0%) |  |
| Grade 2 | 308026 (49.3%) | 456 (22.7%) |  |
| Grade 3 | 82762 (13.2%) | 356 (17.7%) |  |
| Grade 4 | 15944 (2.6%) | 58 (2.9%) |  |
| Other/unknown | 157915 (25.3%) | 1075 (53.6%) |  |
| **Clinical T stage** |  |  | **<0.001** |
| T0 | 3610 (0.6%) | 48 (2.4%) |  |
| T1 | 60393 (9.7%) | 102 (5.1%) |  |
| T2 | 22547 (3.6%) | 24 (1.2%) |  |
| T3 | 61319 (9.8%) | 151 (7.5%) |  |
| T4 | 29911 (4.8%) | 142 (7.1%) |  |
| Other/unknown | 447304 (71.6%) | 1539 (76.7%) |  |
| **Clinical N stage** |  |  | **<0.001** |
| N0 | 349671 (55.9%) | 639 (31.9%) |  |
| N1 | 50169 (8.0%) | 327 (16.3%) |  |
| N2 | 16936 (2.7%) | 154 (7.7%) |  |
| N3 | 0 (0.0%) | 0 (0.0%) |  |
| Other/unknown | 208308 (33.3%) | 886 (44.2%) |  |
| **Bone metastases at diagnosis** |  |  | **<0.001** |
| No | 618346 (98.9%) | 1511 (75.3%) |  |
| Yes | 6401 (1.0%) | 458 (22.8%) |  |
| Other/unknown | 337 (0.1%) | 37 (1.8%) |  |
| **Lung metastases at diagnosis** |  |  | **<0.001** |
| No | 597815 (95.6%) | 943 (47.0%) |  |
| Yes | 25939 (4.1%) | 1021 (50.9%) |  |
| Other/unknown | 1330 (0.2%) | 42 (2.1%) |  |
| **Liver metastases at diagnosis** |  |  | **<0.001** |
| No | 530778 (84.9%) | 883 (44.0%) |  |
| Yes | 93730 (15.0%) | 1085 (54.1%) |  |
| Other/unknown | 576 (0.1%) | 38 (1.9%) |  |
| **CEA (SSF1)** |  |  | **<0.001** |
| Positive | 151256 (24.2%) | 917 (45.7%) |  |
| Negative | 161487 (25.8%) | 175 (8.7%) |  |
| Other/unknown | 312341 (50.0%) | 914 (45.6%) |  |
| **Tumor histology** |  |  | 0.259 |
| Type 1 | 578404 (92.5%) | 1870 (93.2%) |  |
| Type 2 | 46680 (7.5%) | 136 (6.8%) |  |

For histology,

Type 1 = Adenocarcinoma + carcinoma + tubulovillous adenocarcinoma + mucinous adenocarcinoma

Type 2 = Neuroendocrine + Other/unknown

**Supplementary Table 4 - Kidney Cancer Data Stratified by Presence or Absence of Brain Metastasis at Diagnosis**

|  | **Absence of brain metastases**  **(N=402508)** | **Presence of brain metastases**  **(N=5119)** | **P-value** |
| --- | --- | --- | --- |
| **Patient age** |  |  | **0.025** |
| Mean (SD) | 62.9 (13.9) | 63.2 (11.4) |  |
| Median [Min, Max] | 64.0 [0, 90.0] | 63.0 [0, 90.0] |  |
| **Patient sex** |  |  | **<0.001** |
| Male | 251582 (62.5%) | 3532 (69.0%) |  |
| Female | 150926 (37.5%) | 1587 (31.0%) |  |
| **Patient race** |  |  | **<0.001** |
| White | 335328 (83.3%) | 4456 (87.0%) |  |
| Black | 48095 (11.9%) | 389 (7.6%) |  |
| Other | 15377 (3.8%) | 223 (4.4%) |  |
| Unknown | 3708 (0.9%) | 51 (1.0%) |  |
| **Tumor grade** |  |  | **<0.001** |
| Grade 1 | 28853 (7.2%) | 74 (1.4%) |  |
| Grade 2 | 115228 (28.6%) | 341 (6.7%) |  |
| Grade 3 | 69159 (17.2%) | 650 (12.7%) |  |
| Grade 4 | 25247 (6.3%) | 387 (7.6%) |  |
| Other/unknown | 164021 (40.7%) | 3667 (71.6%) |  |
| **Clinical T stage** |  |  | **<0.001** |
| T0 | 744 (0.2%) | 33 (0.6%) |  |
| T1 | 201935 (50.2%) | 896 (17.5%) |  |
| T2 | 39450 (9.8%) | 1156 (22.6%) |  |
| T3 | 28475 (7.1%) | 801 (15.6%) |  |
| T4 | 5286 (1.3%) | 347 (6.8%) |  |
| Other/unknown | 126618 (31.5%) | 1886 (36.8%) |  |
| **Clinical N stage** |  |  | **<0.001** |
| N0 | 289088 (71.8%) | 2277 (44.5%) |  |
| N1 | 16132 (4.0%) | 1055 (20.6%) |  |
| N2 | 1434 (0.4%) | 24 (0.5%) |  |
| N3 | 93 (0.0%) | 1 (0.0%) |  |
| Other/unknown | 95761 (23.8%) | 1762 (34.4%) |  |
| **Bone metastases at diagnosis** |  |  | **<0.001** |
| No | 384554 (95.5%) | 3203 (62.6%) |  |
| Yes | 17734 (4.4%) | 1852 (36.2%) |  |
| Other/unknown | 220 (0.1%) | 64 (1.3%) |  |
| **Lung metastases at diagnosis** |  |  | **<0.001** |
| No | 377473 (93.8%) | 1749 (34.2%) |  |
| Yes | 24558 (6.1%) | 3267 (63.8%) |  |
| Other/unknown | 477 (0.1%) | 103 (2.0%) |  |
| **Liver metastases at diagnosis** |  |  | **<0.001** |
| No | 393218 (97.7%) | 4104 (80.2%) |  |
| Yes | 9013 (2.2%) | 912 (17.8%) |  |
| Other/unknown | 277 (0.1%) | 103 (2.0%) |  |
| **Sarcomatoid Features (SSF4)** |  |  | **<0.001** |
| Yes | 11022 (2.7%) | 369 (7.2%) |  |
| No | 256285 (63.7%) | 1285 (25.1%) |  |
| Other/unknown | 135201 (33.6%) | 3465 (67.7%) |  |
| **Fuhrman Nuclear Grade (SSF6)** |  |  | **<0.001** |
| 1 | 28098 (7.0%) | 73 (1.4%) |  |
| 2 | 125522 (31.2%) | 365 (7.1%) |  |
| 3 | 69357 (17.2%) | 520 (10.2%) |  |
| 4 | 19105 (4.7%) | 440 (8.6%) |  |
| Other/unknown | 160426 (39.9%) | 3721 (72.7%) |  |
| **Tumor histology** |  |  | **<0.001** |
| Type 1 | 71382 (17.7%) | 241 (4.7%) |  |
| Type 2 | 283571 (70.5%) | 4440 (86.7%) |  |
| Type 3 | 47555 (11.8%) | 438 (8.6%) |  |

For histology,

Type 1 = Adenocarcinoma + papillary adenocarcinoma

Type 2 = Renal cell carcinoma

Type 3 = Urothelial cell carcinoma + Other/unknown

**Supplementary Table 5 - Non-Small Cell Lung Cancer Data Stratified by Presence or Absence of Brain Metastasis at Diagnosis**

|  | **Absence of brain metastases**  **(N=950988)** | **Presence of brain metastases**  **(N=109786)** | **P-value** |
| --- | --- | --- | --- |
| **Patient age** |  |  | **<0.001** |
| Mean (SD) | 69.3 (10.5) | 64.9 (10.5) |  |
| Median [Min, Max] | 70.0 [0, 90.0] | 65.0 [0, 90.0] |  |
| **Patient sex** |  |  | **0.004** |
| Male | 485140 (51.0%) | 56507 (51.5%) |  |
| Female | 465848 (49.0%) | 53279 (48.5%) |  |
| **Patient race** |  |  | **<0.001** |
| White | 809790 (85.2%) | 89701 (81.7%) |  |
| Black | 102781 (10.8%) | 13954 (12.7%) |  |
| Other | 32509 (3.4%) | 5331 (4.9%) |  |
| Unknown | 5908 (0.6%) | 800 (0.7%) |  |
| **Tumor grade** |  |  | **<0.001** |
| Grade 1 | 69113 (7.3%) | 1429 (1.3%) |  |
| Grade 2 | 188918 (19.9%) | 8439 (7.7%) |  |
| Grade 3 | 219265 (23.1%) | 26105 (23.8%) |  |
| Grade 4 | 8358 (0.9%) | 1119 (1.0%) |  |
| Other/unknown | 465334 (48.9%) | 72694 (66.2%) |  |
| **Clinical T stage** |  |  | **<0.001** |
| T0 | 3882 (0.4%) | 810 (0.7%) |  |
| T1 | 277274 (29.2%) | 13474 (12.3%) |  |
| T2 | 210569 (22.1%) | 26128 (23.8%) |  |
| T3 | 114159 (12.0%) | 16349 (14.9%) |  |
| T4 | 126884 (13.3%) | 23700 (21.6%) |  |
| Other/unknown | 218220 (22.9%) | 29325 (26.7%) |  |
| **Clinical N stage** |  |  | **<0.001** |
| N0 | 424907 (44.7%) | 20322 (18.5%) |  |
| N1 | 61140 (6.4%) | 8593 (7.8%) |  |
| N2 | 204556 (21.5%) | 38768 (35.3%) |  |
| N3 | 80121 (8.4%) | 17738 (16.2%) |  |
| Other/unknown | 180264 (19.0%) | 24365 (22.2%) |  |
| **Bone metastases at diagnosis** |  |  | **<0.001** |
| No | 835878 (87.9%) | 71765 (65.4%) |  |
| Yes | 113676 (12.0%) | 36811 (33.5%) |  |
| Other/unknown | 1434 (0.2%) | 1210 (1.1%) |  |
| **Lung metastases at diagnosis** |  |  | **<0.001** |
| No | 857556 (90.2%) | 83283 (75.9%) |  |
| Yes | 88557 (9.3%) | 23739 (21.6%) |  |
| Other/unknown | 4875 (0.5%) | 2764 (2.5%) |  |
| **Liver metastases at diagnosis** |  |  | **<0.001** |
| No | 898351 (94.5%) | 89411 (81.4%) |  |
| Yes | 50854 (5.3%) | 18596 (16.9%) |  |
| Other/unknown | 1783 (0.2%) | 1779 (1.6%) |  |
| **Tumor histology** |  |  | **<0.001** |
| Adenocarcinoma | 458542 (48.2%) | 71802 (65.4%) |  |
| Squamous Cell Carcinoma | 282818 (29.7%) | 14197 (12.9%) |  |
| Other/unknown | 209628 (22.0%) | 23787 (21.7%) |  |

**Supplementary Table 6 - Small Cell Lung Cancer Data Stratified by Presence or Absence of Brain Metastasis at Diagnosis**

|  | **Absence of brain metastases**  **(N=138505)** | **Presence of brain metastases**  **(N=26359)** | **P-value** |
| --- | --- | --- | --- |
| **Patient age** |  |  | **<0.001** |
| Mean (SD) | 67.9 (9.88) | 65.4 (9.42) |  |
| Median [Min, Max] | 68.0 [19.0, 90.0] | 65.0 [26.0, 90.0] |  |
| **Patient sex** |  |  | **<0.001** |
| Male | 64756 (46.8%) | 13253 (50.3%) |  |
| Female | 73749 (53.2%) | 13106 (49.7%) |  |
| **Patient race** |  |  | **<0.001** |
| White | 124116 (89.6%) | 23325 (88.5%) |  |
| Black | 10803 (7.8%) | 2316 (8.8%) |  |
| Other | 2805 (2.0%) | 541 (2.1%) |  |
| Unknown | 781 (0.6%) | 177 (0.7%) |  |
| **Tumor grade** |  |  | **<0.001** |
| Grade 1 | 216 (0.2%) | 36 (0.1%) |  |
| Grade 2 | 464 (0.3%) | 62 (0.2%) |  |
| Grade 3 | 11589 (8.4%) | 1934 (7.3%) |  |
| Grade 4 | 16792 (12.1%) | 2668 (10.1%) |  |
| Other/unknown | 109444 (79.0%) | 21659 (82.2%) |  |
| **Clinical T stage** |  |  | **<0.001** |
| T0 | 1330 (1.0%) | 243 (0.9%) |  |
| T1 | 20409 (14.7%) | 2704 (10.3%) |  |
| T2 | 27847 (20.1%) | 5075 (19.3%) |  |
| T3 | 19389 (14.0%) | 3544 (13.4%) |  |
| T4 | 32613 (23.5%) | 6734 (25.5%) |  |
| Other/unknown | 36917 (26.7%) | 8059 (30.6%) |  |
| **Clinical N stage** |  |  | **<0.001** |
| N0 | 19568 (14.1%) | 3081 (11.7%) |  |
| N1 | 10044 (7.3%) | 1803 (6.8%) |  |
| N2 | 59539 (43.0%) | 10892 (41.3%) |  |
| N3 | 23373 (16.9%) | 4488 (17.0%) |  |
| Other/unknown | 25981 (18.8%) | 6095 (23.1%) |  |
| **Bone metastases at diagnosis** |  |  | **<0.001** |
| No | 110485 (79.8%) | 18594 (70.5%) |  |
| Yes | 27533 (19.9%) | 7434 (28.2%) |  |
| Other/unknown | 487 (0.4%) | 331 (1.3%) |  |
| **Lung metastases at diagnosis** |  |  | **<0.001** |
| No | 123368 (89.1%) | 21418 (81.3%) |  |
| Yes | 13665 (9.9%) | 4178 (15.9%) |  |
| Other/unknown | 1472 (1.1%) | 763 (2.9%) |  |
| **Liver metastases at diagnosis** |  |  | **<0.001** |
| No | 99337 (71.7%) | 17545 (66.6%) |  |
| Yes | 38761 (28.0%) | 8455 (32.1%) |  |
| Other/unknown | 407 (0.3%) | 359 (1.4%) |  |

**Supplementary Table 7 – Nomogram Scores for Breast Cancer**

| **Variable** | **Score** |
| --- | --- |
| **Patient age** |  |
| 90 | 0.0 |
| 10-year decrease in age | 1.7 |
| 0 | 15.0 |
| **Patient sex** |  |
| Male | 0.0 |
| Female | 1.9 |
| **Patient race** |  |
| Other | 0.0 |
| Unknown | 4.9 |
| White | 7.4 |
| Black | 8.1 |
| **Tumor grade** |  |
| Grade 1 | 0.0 |
| Grade 2 | 18.7 |
| Grade 3 | 24.7 |
| Grade 4 | 35.7 |
| Other/unknown | 39.9 |
| **Clinical T stage** |  |
| T1 | 0.0 |
| Other/unknown | 7.7 |
| T2 | 11.8 |
| T3 | 19.8 |
| T4 | 20.7 |
| T0 | 30.9 |
| **Clinical N stage** |  |
| N0 | 0.0 |
| N2 | 32.5 |
| N1 | 32.8 |
| Other/unknown | 35.8 |
| N3 | 39.5 |
| **Bone metastases at diagnosis** |  |
| No | 0.0 |
| Other/unknown | 97.6 |
| Yes | 100.0 |
| **Lung metastases at diagnosis** |  |
| No | 0.0 |
| Other/unknown | 58.9 |
| Yes | 62.8 |
| **Liver metastases at diagnosis** |  |
| No | 0.0 |
| Yes | 26.1 |
| Other/unknown | 47.6 |
| **ER (SSF1)** |  |
| Other/unknown | 0.0 |
| Yes | 2.2 |
| No | 20.6 |
| **PR (SSF2)** |  |
| Yes | 0.0 |
| Other/unknown | 11.9 |
| No | 14.8 |
| **HER2 (SSF15)** |  |
| Other/unknown | 0.0 |
| No | 13.9 |
| Yes | 18.5 |

**Supplementary Table 8 – Total Nomogram Scores for Breast Cancer Brain Metastasis Risk**

| **Total Score** | 167.0 | 228.9 | 256.9 | 287.3 | 307.5 | 324.0 | 339.2 | 354.4 | 371.0 | 391.2 | 421.6 |
| --- | --- | --- | --- | --- | --- | --- | --- | --- | --- | --- | --- |
| **Pred. Risk** | 0.01 | 0.05 | 0.1 | 0.2 | 0.3 | 0.4 | 0.5 | 0.6 | 0.7 | 0.8 | 0.9 |

**Supplementary Table 9 – Nomogram Scores for Melanoma**

| **Variable** | **Score** |
| --- | --- |
| **Patient age** |  |
| 90 | 0.0 |
| 10-year decrease in age | 4.0 |
| 0 | 36.3 |
| **Patient sex** |  |
| Female | 0.0 |
| Male | 13.1 |
| **Patient race** |  |
| Black | 0.0 |
| Unknown | 15.8 |
| Other | 19.0 |
| White | 19.0 |
| **Tumor grade** |  |
| Grade 1 | 0.0 |
| Grade 4 | 2.1 |
| Other/unknown | 5.4 |
| Grade 2 | 14.8 |
| Grade 3 | 30.8 |
| **Clinical T stage** |  |
| T1 | 0.0 |
| T2 | 13.0 |
| T3 | 26.8 |
| Other/unknown | 45.5 |
| T4 | 50.4 |
| T0 | 94.0 |
| **Clinical N stage** |  |
| N0 | 0.0 |
| Other/unknown | 11.6 |
| N1 | 14.0 |
| N2 | 14.7 |
| N3 | 22.0 |
| **Bone metastases at diagnosis** |  |
| No | 0.0 |
| Yes | 22.1 |
| Other/unknown | 40.7 |
| **Lung metastases at diagnosis** |  |
| No | 0.0 |
| Other/unknown | 98.9 |
| Yes | 100.0 |
| **Liver metastases at diagnosis** |  |
| No | 0.0 |
| Yes | 16.4 |
| Other/unknown | 46.4 |
| **Ulceration (SSF2)** |  |
| No | 0.0 |
| Yes | 24.9 |
| Other/unknown | 51.0 |

**Supplementary Table 10 – Total Nomogram Scores for Melanoma Brain Metastasis Risk**

| **Total Score** | 126.4 | 178.8 | 202.5 | 228.2 | 245.3 | 259.3 | 272.1 | 285.0 | 299.0 | 316.1 | 341.8 |
| --- | --- | --- | --- | --- | --- | --- | --- | --- | --- | --- | --- |
| **Pred. Risk** | 0.01 | 0.05 | 0.1 | 0.2 | 0.3 | 0.4 | 0.5 | 0.6 | 0.7 | 0.8 | 0.9 |

**Supplementary Table 11 – Nomogram Scores for Colorectal Cancer**

| **Variable** | **Score** |
| --- | --- |
| **Patient age** |  |
| 90 | 0.0 |
| 10-year decrease in age | 3.2 |
| 0 | 28.5 |
| **Patient sex** |  |
| Female | 0.0 |
| Male | 0.7 |
| **Patient race** |  |
| Black | 0.0 |
| Unknown | 1.3 |
| Other | 3.7 |
| White | 13.6 |
| **Tumor grade** |  |
| Grade 1 | 0.0 |
| Grade 2 | 7.4 |
| Grade 3 | 43.6 |
| Grade 4 | 43.8 |
| Other/unknown | 47.2 |
| **Clinical T stage** |  |
| T2 | 0.0 |
| T1 | 9.9 |
| T4 | 10.3 |
| T3 | 11.5 |
| Other/unknown | 17.1 |
| T0 | 61.7 |
| **Clinical N stage** |  |
| N0 | 0.0 |
| Other/unknown | 9.0 |
| N1 | 17.9 |
| N2 | 30.2 |
| N3 | NA |
| **Bone metastases at diagnosis** |  |
| No | 0.0 |
| Yes | 73.9 |
| Other/unknown | 85.8 |
| **Lung metastases at diagnosis** |  |
| No | 0.0 |
| Other/unknown | 65.7 |
| Yes | 100.0 |
| **Liver metastases at diagnosis** |  |
| No | 0.0 |
| Yes | 15.1 |
| Other/unknown | 73.2 |
| **CEA (SSF1)** |  |
| Negative | 0.0 |
| Other/unknown | 15.5 |
| Positive | 26.5 |
| **Tumor histology** |  |
| Type 2 | 0.0 |
| Type 1 | 0.8 |

For histology,

Type 1 = Adenocarcinoma + carcinoma + tubulovillous adenocarcinoma + mucinous adenocarcinoma

Type 2 = Neuroendocrine + other

**Supplementary Table 12 – Total Nomogram Scores for Colorectal Cancer Brain Metastasis Risk**

| **Total Score** | 171.0 | 243.4 | 276.2 | 311.8 | 335.4 | 354.8 | 372.6 | 390.4 | 409.8 | 433.5 | 469.0 |
| --- | --- | --- | --- | --- | --- | --- | --- | --- | --- | --- | --- |
| **Pred. Risk** | 0.01 | 0.05 | 0.1 | 0.2 | 0.3 | 0.4 | 0.5 | 0.6 | 0.7 | 0.8 | 0.9 |

**Supplementary Table 13 – Nomogram Scores for Kidney Cancer**

| **Variable** | **Score** |
| --- | --- |
| **Patient age** |  |
| 90 | 0.0 |
| 10-year decrease in age | 3.2 |
| 0 | 28.6 |
| **Patient sex** |  |
| Female | 0.0 |
| Male | 5.3 |
| **Patient race** |  |
| Black | 0.0 |
| White | 14.4 |
| Other | 15.8 |
| Unknown | 16.8 |
| **Tumor grade** |  |
| Grade 1 | 0.0 |
| Grade 2 | 6.7 |
| Grade 4 | 7.1 |
| Grade 3 | 16.7 |
| Other/unknown | 20.2 |
| **Clinical T stage** |  |
| T1 | 0.0 |
| Other/unknown | 7.5 |
| T3 | 20.8 |
| T4 | 23.1 |
| T0 | 33.9 |
| T2 | 34.1 |
| **Clinical N stage** |  |
| N3 | 0.0 |
| N2 | 31.3 |
| N0 | 43.8 |
| Other/unknown | 46.5 |
| N1 | 49.6 |
| **Bone metastases at diagnosis** |  |
| No | 0.0 |
| Yes | 31.5 |
| Other/unknown | 48.0 |
| **Lung metastases at diagnosis** |  |
| No | 0.0 |
| Yes | 92.3 |
| Other/unknown | 100.0 |
| **Liver metastases at diagnosis** |  |
| No | 0.0 |
| Yes | 8.9 |
| Other/unknown | 38.0 |
| **Sarcomatoid Features (SSF4)** |  |
| No | 0.0 |
| Yes | 11.9 |
| Other/unknown | 23.8 |
| **Fuhrman Nuclear Grade (SSF6)** |  |
| 2 | 0.0 |
| 1 | 5.4 |
| 3 | 8.7 |
| 4 | 20.5 |
| Other/unknown | 23.4 |
| **Tumor histology** |  |
| Type 1 | 0.0 |
| Type 3 | 3.7 |
| Type 2 | 36.5 |

For histology,

Type 1 = Adenocarcinoma + papillary adenocarcinoma

Type 2 = Renal cell carcinoma

Type 3 = Urothelial cell carcinoma + other

**Supplementary Table 14 – Total Nomogram Scores for Kidney Cancer Brain Metastasis Risk**

| **Total Score** | 179.9 | 244.7 | 274.1 | 305.9 | 327.1 | 344.4 | 360.4 | 376.3 | 393.6 | 414.8 | 446.7 |
| --- | --- | --- | --- | --- | --- | --- | --- | --- | --- | --- | --- |
| **Pred. Risk** | 0.01 | 0.05 | 0.1 | 0.2 | 0.3 | 0.4 | 0.5 | 0.6 | 0.7 | 0.8 | 0.9 |

**Supplementary Table 15 – Nomogram Scores for Non-Small Cell Lung Cancer**

| **Variable** | **Score** |
| --- | --- |
| **Patient age** |  |
| 90 | 0.0 |
| 10-year decrease in age | 11.1 |
| 0 | 100.0 |
| **Patient sex** |  |
| Male | 0.0 |
| Female | 0.4 |
| **Patient race** |  |
| White | 0.0 |
| Black | 0.0 |
| Unknown | 4.0 |
| Other | 4.9 |
| **Tumor grade** |  |
| Grade 1 | 0.0 |
| Grade 2 | 25.2 |
| Grade 3 | 46.5 |
| Grade 4 | 47.2 |
| Other/unknown | 50.6 |
| **Clinical T stage** |  |
| T1 | 0.0 |
| Other/unknown | 12.4 |
| T4 | 18.5 |
| T3 | 18.8 |
| T2 | 20.4 |
| T0 | 25.1 |
| **Clinical N stage** |  |
| N0 | 0.0 |
| Other/unknown | 17.4 |
| N1 | 22.4 |
| N3 | 24.2 |
| N2 | 25.6 |
| **Bone metastases at diagnosis** |  |
| No | 0.0 |
| Yes | 21.9 |
| Other/unknown | 38.6 |
| **Lung metastases at diagnosis** |  |
| No | 0.0 |
| Yes | 14.4 |
| Other/unknown | 27.1 |
| **Liver metastases at diagnosis** |  |
| No | 0.0 |
| Yes | 19.5 |
| Other/unknown | 39.3 |
| **Tumor histology** |  |
| Squamous Cell Carcinoma | 0.0 |
| Other | 24.9 |
| Adenocarcinoma | 34.4 |

**Supplementary Table 16 – Total Nomogram Scores for Non-Small Cell Lung Cancer Brain Metastasis Risk**

| **Total Score** | 51.9 | 107.9 | 133.3 | 160.9 | 179.2 | 194.2 | 208.0 | 221.7 | 236.8 | 255.1 | 282.6 |
| --- | --- | --- | --- | --- | --- | --- | --- | --- | --- | --- | --- |
| **Pred. Risk** | 0.01 | 0.05 | 0.1 | 0.2 | 0.3 | 0.4 | 0.5 | 0.6 | 0.7 | 0.8 | 0.9 |

**Supplementary Table 17 – Nomogram Scores for Small Cell Lung Cancer**

| **Variable** | **Score** |
| --- | --- |
| **Patient age** |  |
| 90 | 0.0 |
| 10-year decrease in age | 11.1 |
| 0 | 100.0 |
| **Patient sex** |  |
| Female | 0.0 |
| Male | 4.8 |
| **Patient race** |  |
| White | 0.0 |
| Other | 0.3 |
| Black | 4.6 |
| Unknown | 8.0 |
| **Tumor grade** |  |
| Grade 2 | 0.0 |
| Grade 4 | 2.2 |
| Grade 3 | 5.0 |
| Grade 1 | 6.8 |
| Other/unknown | 9.2 |
| **Clinical T stage** |  |
| T1 | 0.0 |
| T3 | 8.8 |
| Other/unknown | 9.7 |
| T0 | 10.1 |
| T4 | 10.5 |
| T2 | 10.9 |
| **Clinical N stage** |  |
| N3 | 0.0 |
| N2 | 1.7 |
| N0 | 2.0 |
| N1 | 4.1 |
| Other/unknown | 10.4 |
| **Bone metastases at diagnosis** |  |
| No | 0.0 |
| Yes | 15.5 |
| Other/unknown | 33.0 |
| **Lung metastases at diagnosis** |  |
| No | 0.0 |
| Yes | 21.5 |
| Other/unknown | 32.3 |
| **Liver metastases at diagnosis** |  |
| No | 0.0 |
| Yes | 0.2 |
| Other/unknown | 39.9 |

**Supplementary Table 18 – Total Nomogram Scores for Small Cell Lung Cancer Brain Metastasis Risk**

| **Total Score** | 33.5 | 68.3 | 91.4 | 110.3 | 127.7 | 145.1 | 164.0 | 187.2 | 221.9 |
| --- | --- | --- | --- | --- | --- | --- | --- | --- | --- |
| **Pred. Risk** | 0.1 | 0.2 | 0.3 | 0.4 | 0.5 | 0.6 | 0.7 | 0.8 | 0.9 |

**Supplementary Table 19 – Characteristics of Patients with Breast Cancer at Low, Intermediate, and High Risk of Brain Metastasis**

| **Risk of BM** | **<1% (N=2030321)** | **1%~10% (N=50130)** | **>10% (N=14888)** |
| --- | --- | --- | --- |
| **Patient age** |  |  |  |
| Mean (SD) | 61.33 (13.02) | 61.98 (14.06) | 61.55 (13.40) |
| Median [Min, Max] | 62 [0,90] | 62 [0,90] | 62 [15,90] |
| **Patient sex** |  |  |  |
| Male | 16877 (0.8%) | 718 (1.4%) | 234 (1.6%) |
| Female | 2013444 (99.2%) | 49412 (98.6%) | 14654 (98.4%) |
| **Patient race** |  |  |  |
| White | 1664131 (82%) | 39189 (78.2%) | 11114 (74.7%) |
| Black | 238470 (11.7%) | 8390 (16.7%) | 3021 (20.3%) |
| Other | 108936 (5.4%) | 2129 (4.2%) | 631 (4.2%) |
| Unknown | 18784 (0.9%) | 422 (0.8%) | 122 (0.8%) |
| **Tumor grade** |  |  |  |
| Grade 1 | 354987 (17.5%) | 2171 (4.3%) | 136 (0.9%) |
| Grade 2 | 719031 (35.4%) | 13734 (27.4%) | 3181 (21.4%) |
| Grade 3 | 517950 (25.5%) | 14866 (29.7%) | 5155 (34.6%) |
| Grade 4 | 7794 (0.4%) | 184 (0.4%) | 87 (0.6%) |
| Other/unknown | 430559 (21.2%) | 19175 (38.3%) | 6329 (42.5%) |
| **Clinical T stage** |  |  |  |
| T1 | 820224 (40.4%) | 4455 (8.9%) | 583 (3.9%) |
| T0 | 8359 (0.4%) | 1262 (2.5%) | 290 (1.9%) |
| T2 | 347609 (17.1%) | 11444 (22.8%) | 2426 (16.3%) |
| T3 | 65219 (3.2%) | 6292 (12.6%) | 1784 (12.0%) |
| T4 | 40061 (2.0%) | 11885 (23.7%) | 5564 (37.4%) |
| Other/unknown | 748849 (36.9%) | 14792 (29.5%) | 4241 (28.5%) |
| **Clinical N stage** |  |  |  |
| N0 | 1459185 (71.9%) | 8659 (17.3%) | 906 (6.1%) |
| N1 | 173921 (8.6%) | 18504 (36.9%) | 5696 (38.3%) |
| N2 | 26434 (1.3%) | 4526 (9%) | 1813 (12.2%) |
| N3 | 16519 (0.8%) | 4634 (9.2%) | 2243 (15.1%) |
| Other/unknown | 354262 (17.4%) | 13807 (27.5%) | 4230 (28.4%) |
| **Bone metastases at diagnosis** |  |  |  |
| No | 2025484 (99.8%) | 9385 (18.7%) | 10 (0.1%) |
| Yes | 4805 (0.2%) | 40560 (80.9%) | 14641 (98.3%) |
| Other/unknown | 32 (0.0%) | 185 (0.4%) | 237 (1.6%) |
| **Lung metastases at diagnosis** |  |  |  |
| No | 2026562 (99.8%) | 39061 (77.9%) | 1717 (11.5%) |
| Yes | 3651 (0.2%) | 10723 (21.4%) | 12467 (83.7%) |
| Other/unknown | 108 (0.0%) | 346 (0.7%) | 704 (4.7%) |
| **Liver metastases at diagnosis** |  |  |  |
| No | 2024634 (99.7%) | 40495 (80.8%) | 7555 (50.7%) |
| Yes | 5646 (0.3%) | 9421 (18.8%) | 6868 (46.1%) |
| Other/unknown | 41 (0.0%) | 214 (0.4%) | 465 (3.1%) |
| **ER (SSF1)** |  |  |  |
| Yes | 1418021 (69.8%) | 31219 (62.3%) | 7755 (52.1%) |
| No | 283775 (14%) | 8964 (17.9%) | 4191 (28.2%) |
| Other/unknown | 328525 (16.2%) | 9947 (19.8%) | 2942 (19.8%) |
| **PR (SSF2)** |  |  |  |
| Yes | 1235530 (60.9%) | 24882 (49.6%) | 5985 (40.2%) |
| No | 442463 (21.8%) | 14849 (29.6%) | 5821 (39.1%) |
| Other/unknown | 352328 (17.4%) | 10399 (20.7%) | 3082 (20.7%) |
| **HER2 (SSF15)** |  |  |  |
| Yes | 204044 (10%) | 9220 (18.4%) | 3668 (24.6%) |
| No | 1165682 (57.4%) | 28539 (56.9%) | 7719 (51.8%) |
| Other/unknown | 660595 (32.5%) | 12371 (24.7%) | 3501 (23.5%) |

**Supplementary Table 20 – Characteristics of Patients with Melanoma at Low, Intermediate, and High Risk of Brain Metastasis**

| **Risk of BM** | **<1% (N=367207)** | **1%~10% (N=95581)** | **>10% (N=9823)** |
| --- | --- | --- | --- |
| **Patient age** |  |  |  |
| Mean (SD) | 62.98 (15.58) | 63.19 (15.09) | 64.38 (14.56) |
| Median [Min, Max] | 65 [0,90] | 65 [0,90] | 66 [0,90] |
| **Patient sex** |  |  |  |
| Male | 209205 (57%) | 59005 (61.7%) | 7172 (73%) |
| Female | 158002 (43%) | 36576 (38.3%) | 2651 (27%) |
| **Patient race** |  |  |  |
| White | 358367 (97.6%) | 93040 (97.3%) | 9551 (97.2%) |
| Black | 1993 (0.5%) | 345 (0.4%) | 105 (1.1%) |
| Other | 2962 (0.8%) | 1028 (1.1%) | 102 (1.0%) |
| Unknown | 3885 (1.1%) | 1168 (1.2%) | 65 (0.7%) |
| **Tumor grade** |  |  |  |
| Grade 1 | 1070 (0.3%) | 86 (0.1%) | 6 (0.1%) |
| Grade 2 | 573 (0.2%) | 59 (0.1%) | 1 (0.0%) |
| Grade 3 | 691 (0.2%) | 443 (0.5%) | 208 (2.1%) |
| Grade 4 | 447 (0.1%) | 58 (0.1%) | 29 (0.3%) |
| Other/unknown | 364426 (99.2%) | 94935 (99.3%) | 9579 (97.5%) |
| **Clinical T stage** |  |  |  |
| T1 | 127302 (34.7%) | 156 (0.2%) | 23 (0.2%) |
| T0 | 108 (0%) | 2652 (2.8%) | 3211 (32.7%) |
| T2 | 49094 (13.4%) | 175 (0.2%) | 36 (0.4%) |
| T3 | 27193 (7.4%) | 444 (0.5%) | 146 (1.5%) |
| T4 | 14757 (4%) | 4151 (4.3%) | 600 (6.1%) |
| Other/unknown | 148753 (40.5%) | 88003 (92.1%) | 5807 (59.1%) |
| **Clinical N stage** |  |  |  |
| N0 | 329150 (89.6%) | 20797 (21.8%) | 2380 (24.2%) |
| N1 | 4517 (1.2%) | 3017 (3.2%) | 1077 (11%) |
| N2 | 1829 (0.5%) | 1198 (1.3%) | 379 (3.9%) |
| N3 | 842 (0.2%) | 1287 (1.3%) | 605 (6.2%) |
| Other/unknown | 30869 (8.4%) | 69282 (72.5%) | 5382 (54.8%) |
| **Bone metastases at diagnosis** |  |  |  |
| No | 366976 (99.9%) | 94305 (98.7%) | 7202 (73.3%) |
| Yes | 228 (0.1%) | 1260 (1.3%) | 2419 (24.6%) |
| Other/unknown | 3 (0.0%) | 16 (0.0%) | 202 (2.1%) |
| **Lung metastases at diagnosis** |  |  |  |
| No | 367205 (100%) | 94814 (99.2%) | 1431 (14.6%) |
| Yes | 1 (0.0%) | 750 (0.8%) | 8120 (82.7%) |
| Other/unknown | 1 (0.0%) | 17 (0.0%) | 272 (2.8%) |
| **Liver metastases at diagnosis** |  |  |  |
| No | 366965 (99.9%) | 94152 (98.5%) | 6782 (69%) |
| Yes | 241 (0.1%) | 1421 (1.5%) | 2829 (28.8%) |
| Other/unknown | 1 (0.0%) | 8 (0.0%) | 212 (2.2%) |
| **Ulceration (SSF2)** |  |  |  |
| Yes | 44937 (12.2%) | 6885 (7.2%) | 855 (8.7%) |
| No | 316669 (86.2%) | 2142 (2.2%) | 845 (8.6%) |
| Other/unknown | 5601 (1.5%) | 86554 (90.6%) | 8123 (82.7%) |

**Supplementary Table 21 – Characteristics of Patients with Colorectal Cancer at Low, Intermediate, and High Risk of Brain Metastasis**

| **Risk of BM** | **<1% (N=598364)** | **1%~10% (N=26474)** | **>10% (N=2252)** |
| --- | --- | --- | --- |
| **Patient age** |  |  |  |
| Mean (SD) | 67.34 (14.12) | 66.03 (13.65) | 64.79 (13.35) |
| Median [Min, Max] | 68 [0,90] | 66 [4,90] | 65 [17,90] |
| **Patient sex** |  |  |  |
| Male | 294261 (49.2%) | 13814 (52.2%) | 1305 (57.9%) |
| Female | 304103 (50.8%) | 12660 (47.8%) | 947 (42.1%) |
| **Patient race** |  |  |  |
| White | 489645 (81.8%) | 20796 (78.6%) | 1784 (79.2%) |
| Black | 76935 (12.9%) | 4255 (16.1%) | 349 (15.5%) |
| Other | 27069 (4.5%) | 1216 (4.6%) | 99 (4.4%) |
| Unknown | 4715 (0.8%) | 207 (0.8%) | 20 (0.9%) |
| **Tumor grade** |  |  |  |
| Grade 1 | 59846 (10.0%) | 637 (2.4%) | 15 (0.7%) |
| Grade 2 | 300853 (50.3%) | 7476 (28.2%) | 153 (6.8%) |
| Grade 3 | 78820 (13.2%) | 3932 (14.9%) | 366 (16.3%) |
| Grade 4 | 15371 (2.6%) | 592 (2.2%) | 39 (1.7%) |
| Other/unknown | 143474 (24%) | 13837 (52.3%) | 1679 (74.6%) |
| **Clinical T stage** |  |  |  |
| T1 | 59206 (9.9%) | 1210 (4.6%) | 79 (3.5%) |
| T0 | 3151 (0.5%) | 432 (1.6%) | 75 (3.3%) |
| T2 | 22344 (3.7%) | 216 (0.8%) | 11 (0.5%) |
| T3 | 59303 (9.9%) | 2051 (7.7%) | 116 (5.2%) |
| T4 | 27465 (4.6%) | 2433 (9.2%) | 155 (6.9%) |
| Other/unknown | 426895 (71.3%) | 20132 (76.0%) | 1816 (80.6%) |
| **Clinical N stage** |  |  |  |
| N0 | 342300 (57.2%) | 7540 (28.5%) | 470 (20.9%) |
| N1 | 44974 (7.5%) | 5065 (19.1%) | 457 (20.3%) |
| N2 | 14826 (2.5%) | 2051 (7.7%) | 213 (9.5%) |
| Other/unknown | 196264 (32.8%) | 11818 (44.6%) | 1112 (49.4%) |
| **Bone metastases at diagnosis** |  |  |  |
| No | 597295 (99.8%) | 22501 (85%) | 61 (2.7%) |
| Yes | 1035 (0.2%) | 3775 (14.3%) | 2049 (91%) |
| Other/unknown | 34 (0.0%) | 198 (0.7%) | 142 (6.3%) |
| **Lung metastases at diagnosis** |  |  |  |
| No | 595381 (99.5%) | 3372 (12.7%) | 5 (0.2%) |
| Yes | 2569 (0.4%) | 22218 (83.9%) | 2173 (96.5%) |
| Other/unknown | 414 (0.1%) | 884 (3.3%) | 74 (3.3%) |
| **Liver metastases at diagnosis** |  |  |  |
| No | 525160 (87.8%) | 6170 (23.3%) | 331 (14.7%) |
| Yes | 73064 (12.2%) | 19937 (75.3%) | 1814 (80.6%) |
| Other/unknown | 140 (0.0%) | 367 (1.4%) | 107 (4.8%) |
| **CEA (SSF1)** |  |  |  |
| Positive | 136069 (22.7%) | 14861 (56.1%) | 1243 (55.2%) |
| Negative | 160344 (26.8%) | 1265 (4.8%) | 53 (2.4%) |
| Other/unknown | 301951 (50.5%) | 10348 (39.1%) | 956 (42.5%) |
| **Tumor Histology** |  |  |  |
| Type 1 | 552962 (92.4%) | 25204 (95.2%) | 2108 (93.6%) |
| Type 2 | 45402 (7.6%) | 1270 (4.8%) | 144 (6.4%) |

For histology,

Type 1 = Adenocarcinoma + carcinoma + tubulovillous adenocarcinoma + mucinous adenocarcinoma

Type 2 = Neuroendocrine + other/unknown

**Supplementary Table 22 – Characteristics of Patients with Kidney Cancer at Low, Intermediate, and High Risk of Brain Metastasis**

| **Risk of BM** | **<1% (N=337199)** | **1%~10% (N=56698)** | **>10% (N=13730)** |
| --- | --- | --- | --- |
| **Patient age** |  |  |  |
| Mean (SD) | 63.06 (13.83) | 62.11 (14.29) | 63.68 (12.05) |
| Median [Min, Max] | 64 [0,90] | 63 [0,90] | 63 [0,90] |
| **Patient sex** |  |  |  |
| Male | 205432 (60.9%) | 39706 (70%) | 9976 (72.7%) |
| Female | 131767 (39.1%) | 16992 (30%) | 3754 (27.3%) |
| **Patient race** |  |  |  |
| White | 277504 (82.3%) | 50228 (88.6%) | 12052 (87.8%) |
| Black | 44505 (13.2%) | 3040 (5.4%) | 939 (6.8%) |
| Other | 12173 (3.6%) | 2819 (5%) | 608 (4.4%) |
| Unknown | 3017 (0.9%) | 611 (1.1%) | 131 (1.0%) |
| **Tumor grade** |  |  |  |
| Grade 1 | 28596 (8.5%) | 291 (0.5%) | 40 (0.3%) |
| Grade 2 | 113649 (33.7%) | 1742 (3.1%) | 178 (1.3%) |
| Grade 3 | 63329 (18.8%) | 5226 (9.2%) | 1254 (9.1%) |
| Grade 4 | 20886 (6.2%) | 3872 (6.8%) | 876 (6.4%) |
| Other/unknown | 110739 (32.8%) | 45567 (80.4%) | 11382 (82.9%) |
| **Clinical T stage** |  |  |  |
| T1 | 196287 (58.2%) | 5482 (9.7%) | 1062 (7.7%) |
| T0 | 393 (0.1%) | 280 (0.5%) | 104 (0.8%) |
| T2 | 29299 (8.7%) | 7739 (13.6%) | 3568 (26%) |
| T3 | 20543 (6.1%) | 6161 (10.9%) | 2572 (18.7%) |
| T4 | 2200 (0.7%) | 2097 (3.7%) | 1336 (9.7%) |
| Other/unknown | 88477 (26.2%) | 34939 (61.6%) | 5088 (37.1%) |
| **Clinical N stage** |  |  |  |
| N0 | 270005 (80.1%) | 16561 (29.2%) | 4799 (35%) |
| N1 | 6939 (2.1%) | 6328 (11.2%) | 3920 (28.6%) |
| N2 | 1044 (0.3%) | 379 (0.7%) | 35 (0.3%) |
| N3 | 62 (0.0%) | 31 (0.1%) | 1 (0.0%) |
| Other/unknown | 59149 (17.5%) | 33399 (58.9%) | 4975 (36.2%) |
| **Bone metastases at diagnosis** |  |  |  |
| No | 334301 (99.1%) | 47131 (83.1%) | 6325 (46.1%) |
| Yes | 2881 (0.9%) | 9486 (16.7%) | 7219 (52.6%) |
| Other/unknown | 17 (0.0%) | 81 (0.1%) | 186 (1.4%) |
| **Lung metastases at diagnosis** |  |  |  |
| No | 337159 (100%) | 42060 (74.2%) | 3 (0.0%) |
| Yes | 40 (0.0%) | 14450 (25.5%) | 13335 (97.1%) |
| Other/unknown | 0 (0.0%) | 188 (0.3%) | 392 (2.9%) |
| **Liver metastases at diagnosis** |  |  |  |
| No | 335214 (99.4%) | 52341 (92.3%) | 9767 (71.1%) |
| Yes | 1978 (0.6%) | 4277 (7.5%) | 3670 (26.7%) |
| Other/unknown | 7 (0.0%) | 80 (0.1%) | 293 (2.1%) |
| **Sarcomatoid Features (SSF4)** |  |  |  |
| Yes | 6601 (2.0%) | 3532 (6.2%) | 1258 (9.2%) |
| No | 246971 (73.2%) | 9051 (16%) | 1548 (11.3%) |
| Other/unknown | 83627 (24.8%) | 44115 (77.8%) | 10924 (79.6%) |
| **Fuhrman Nuclear Grade (SSF6)** |  |  |  |
| 1 | 27875 (8.3%) | 261 (0.5%) | 35 (0.3%) |
| 2 | 123893 (36.7%) | 1827 (3.2%) | 167 (1.2%) |
| 3 | 65255 (19.4%) | 3865 (6.8%) | 757 (5.5%) |
| 4 | 13756 (4.1%) | 4578 (8.1%) | 1211 (8.8%) |
| Other/unknown | 106420 (31.6%) | 46167 (81.4%) | 11560 (84.2%) |
| **Tumor Histology** |  |  |  |
| Type 1 | 69669 (20.7%) | 1806 (3.2%) | 148 (1.1%) |
| Type 2 | 224751 (66.7%) | 50365 (88.8%) | 12895 (93.9%) |
| Type 3 | 42779 (12.7%) | 4527 (8.0%) | 687 (5.0%) |

For histology,

Type 1 = Adenocarcinoma + papillary adenocarcinoma

Type 2 = Renal cell carcinoma

Type 3 = Urothelial cell carcinoma + other/unknown

**Supplementary Table 23 – Characteristics of Patients with Non-Small Cell Lung Cancer at Low, Intermediate, and High Risk of Brain Metastasis**

| **Risk of BM** | **<1% (N=33966)** | **1%~10% (N=634488)** | **>10% (N=392320)** |
| --- | --- | --- | --- |
| **Patient age** |  |  |  |
| Mean (SD) | 76.63 (6.54) | 71.23 (9.69) | 64.31 (10.59) |
| Median [Min, Max] | 76 [44,90] | 72 [5,90] | 64 [0,90] |
| **Patient sex** |  |  |  |
| Male | 16156 (47.6%) | 324396 (51.1%) | 201095 (51.3%) |
| Female | 17810 (52.4%) | 310092 (48.9%) | 191225 (48.7%) |
| **Patient race** |  |  |  |
| White | 31063 (91.5%) | 550755 (86.8%) | 317673 (81%) |
| Black | 2285 (6.7%) | 61940 (9.8%) | 52510 (13.4%) |
| Other | 483 (1.4%) | 18105 (2.9%) | 19252 (4.9%) |
| Unknown | 135 (0.4%) | 3688 (0.6%) | 2885 (0.7%) |
| **Tumor grade** |  |  |  |
| Grade 1 | 17006 (50.1%) | 52731 (8.3%) | 805 (0.2%) |
| Grade 2 | 16207 (47.7%) | 166026 (26.2%) | 15124 (3.9%) |
| Grade 3 | 551 (1.6%) | 149973 (23.6%) | 94846 (24.2%) |
| Grade 4 | 4 (0.0%) | 5145 (0.8%) | 4328 (1.1%) |
| Other/unknown | 198 (0.6%) | 260613 (41.1%) | 277217 (70.7%) |
| **Clinical T stage** |  |  |  |
| T1 | 30316 (89.3%) | 230566 (36.3%) | 29866 (7.6%) |
| T0 | 7 (0.0%) | 1395 (0.2%) | 3290 (0.8%) |
| T2 | 1649 (4.9%) | 140694 (22.2%) | 94354 (24.1%) |
| T3 | 703 (2.1%) | 69059 (10.9%) | 60746 (15.5%) |
| T4 | 443 (1.3%) | 62651 (9.9%) | 87490 (22.3%) |
| Other/unknown | 848 (2.5%) | 130123 (20.5%) | 116574 (29.7%) |
| **Clinical N stage** |  |  |  |
| N0 | 33493 (98.6%) | 373248 (58.8%) | 38488 (9.8%) |
| N1 | 112 (0.3%) | 36302 (5.7%) | 33319 (8.5%) |
| N2 | 124 (0.4%) | 90255 (14.2%) | 152945 (39.0%) |
| N3 | 39 (0.1%) | 28726 (4.5%) | 69094 (17.6%) |
| Other/unknown | 198 (0.6%) | 105957 (16.7%) | 98474 (25.1%) |
| **Bone metastases at diagnosis** |  |  |  |
| No | 33948 (99.9%) | 614199 (96.8%) | 259496 (66.1%) |
| Yes | 18 (0.1%) | 20215 (3.2%) | 130254 (33.2%) |
| Other/unknown | 0 (0.0%) | 74 (0.0%) | 2570 (0.7%) |
| **Lung metastases at diagnosis** |  |  |  |
| No | 33880 (99.7%) | 606482 (95.6%) | 300477 (76.6%) |
| Yes | 86 (0.3%) | 27463 (4.3%) | 84747 (21.6%) |
| Other/unknown | 0 (0.0%) | 543 (0.1%) | 7096 (1.8%) |
| **Liver metastases at diagnosis** |  |  |  |
| No | 33956 (100%) | 625826 (98.6%) | 327980 (83.6%) |
| Yes | 10 (0.0%) | 8599 (1.4%) | 60841 (15.5%) |
| Other/unknown | 0 (0.0%) | 63 (0.0%) | 3499 (0.9%) |
| **Tumor Histology** |  |  |  |
| Adenocarcinoma | 6995 (20.6%) | 245711 (38.7%) | 277638 (70.8%) |
| Squamous Cell Carcinoma | 20191 (59.4%) | 251408 (39.6%) | 25416 (6.5%) |
| Other | 6780 (20.0%) | 137369 (21.7%) | 89266 (22.8%) |

**Supplementary Table 24 – Characteristics of Patients with Small Cell Lung Cancer at Low, Intermediate, and High Risk of Brain Metastasis**

| **Risk of BM** | **<1% (N=0)** | **1%~10% (N=17879)** | **>10% (N=146985)** |
| --- | --- | --- | --- |
| **Patient age** |  |  |  |
| Mean (SD) |  | 79.69 (5.76) | 65.97 (9.19) |
| Median [Min, Max] |  | 80 [61,90] | 66 [19,90] |
| **Patient sex** |  |  |  |
| Male |  | 5796 (32.4%) | 72213 (49.1%) |
| Female |  | 12083 (67.6%) | 74772 (50.9%) |
| **Patient race** |  |  |  |
| White |  | 16880 (94.4%) | 130561 (88.8%) |
| Black |  | 641 (3.6%) | 12478 (8.5%) |
| Other |  | 329 (1.8%) | 3017 (2.1%) |
| Unknown |  | 29 (0.2%) | 929 (0.6%) |
| **Tumor grade** |  |  |  |
| Grade 1 |  | 53 (0.3%) | 199 (0.1%) |
| Grade 2 |  | 209 (1.2%) | 317 (0.2%) |
| Grade 3 |  | 2536 (14.2%) | 10987 (7.5%) |
| Grade 4 |  | 4463 (25.0%) | 14997 (10.2%) |
| Other/unknown |  | 10618 (59.4%) | 120485 (82.0%) |
| **Clinical T stage** |  |  |  |
| T1 |  | 8376 (46.8%) | 14737 (10.0%) |
| T0 |  | 136 (0.8%) | 1437 (1.0%) |
| T2 |  | 2859 (16.0%) | 30063 (20.5%) |
| T3 |  | 2317 (13.0%) | 20616 (14.0%) |
| T4 |  | 2393 (13.4%) | 36954 (25.1%) |
| Other/unknown |  | 1798 (10.1%) | 43178 (29.4%) |
| **Clinical N stage** |  |  |  |
| N0 |  | 5142 (28.8%) | 17507 (11.9%) |
| N1 |  | 1435 (8.0%) | 10412 (7.1%) |
| N2 |  | 8167 (45.7%) | 62264 (42.4%) |
| N3 |  | 2713 (15.2%) | 25148 (17.1%) |
| Other/unknown |  | 422 (2.4%) | 31654 (21.5%) |
| **Bone metastases at diagnosis** |  |  |  |
| No |  | 17772 (99.4%) | 111307 (75.7%) |
| Yes |  | 107 (0.6%) | 34860 (23.7%) |
| Other/unknown |  | 0 (0.0%) | 818 (0.6%) |
| **Lung metastases at diagnosis** |  |  |  |
| No |  | 17876 (100%) | 126910 (86.3%) |
| Yes |  | 3 (0.0%) | 17840 (12.1%) |
| Other/unknown |  | 0 (0.0%) | 2235 (1.5%) |
| **Liver metastases at diagnosis** |  |  |  |
| No |  | 14880 (83.2%) | 102002 (69.4%) |
| Yes |  | 2999 (16.8%) | 44217 (30.1%) |
| Other/unknown |  | 0 (0.0%) | 766 (0.5%) |

**Supplementary Table 25 –ROC-AUC Evaluated for Each Cancer Type’s Model Based on 7:3 Random-Split Training/Testing Data**

| **Cancer Type** | **Mean AUC^*^** | **2.5% quantile^*^** | **97.5% quantile^*^** |
| --- | --- | --- | --- |
| **Breast** | 0.9534 | 0.9498 | 0.9567 |
| **Melanoma** | 0.9420 | 0.9378 | 0.9454 |
| **Colorectal** | 0.8785 | 0.8728 | 0.8844 |
| **Kidney** | 0.9054 | 0.8991 | 0.9119 |
| **Non-small cell lung** | 0.7759 | 0.7741 | 0.7781 |
| **Small cell lung** | 0.6180 | 0.6140 | 0.6227 |

*Based on 100 random splits of the data

**Supplementary Table 26 – Supplementary Model Performance Metrics for Each Cancer Type’s Model (Average maximum-attainable metrics based on iteration-wise optimal cutpoints)**

| **Cancer Type** | **Average Optimal Cutpoint** | **Average Overall Accuracy** | **Average Sensitivity** | **Average Specificity** | **Average Positive Predictive Value** | **Average Negative Predictive Value** |
| --- | --- | --- | --- | --- | --- | --- |
| **Breast** | 0.0028 | 92.33% | 88.37% | 92.34% | 3.78% | 99.96% |
| **Melanoma** | 0.0756 | 94.60% | 65.88% | 95.11% | 19.40% | 99.36% |
| **Colorectal** | 0.0048 | 90.20% | 68.44% | 90.27% | 2.22% | 99.89% |
| **Kidney** | 0.0132 | 90.03% | 76.51% | 90.20% | 9.13% | 99.67% |
| **Nonsmall-cell lung** | 0.0935 | 66.03% | 76.69% | 64.80% | 20.11% | 96.01% |
| **Small-cell lung** | 0.1547 | 57.94% | 59.18% | 57.71% | 21.05% | 88.15% |

**Supplementary Table 27 – Patients with brain metastasis stratified by presence or absence of metastatic disease to liver, lung, or bone.**

| **Cancer Type**  **(total number of patients with brain metastases)** | **Brain metastasis with liver, lung, or bone metastasis (or other/unknown)** | **No liver, lung, or bone metastases but with brain metastases** |
| --- | --- | --- |
| **Breast**  **(n=7077)** | 5804 (82.01%) | 1273 (17.99%) |
| **Melanoma**  **(n=6912)** | 4388 (62.76%) | 2574 (37.24%) |
| **Colorectal**  **(n=2006)** | 1532 (76.37%) | 474 (23.63%) |
| **Kidney**  **(n=5119)** | 3961 (77.38%) | 1158 (22.62%) |
| **Non-small cell lung**  **(n=109786)** | 56299 (51.28%) | 53487 (48.72%) |
| **Small cell lung**  **(n=26359)** | 13774 (52.26%) | 12585 (47.74%) |
