# Supplementary material for: Estimating the risk of brain metastasis for patients newly diagnosed with cancer
Source: Commun Med (Lond). 2024 Feb 22;4:27. doi: 10.1038/s43856-024-00445-7 (PMC10883934; doi:10.1038/s43856-024-00445-7)
Supplement: Supplementary file 3 — Description of Additional Supplementary Files [file 43856_2024_445_MOESM3_ESM.docx]

**Description of Additional Supplementary Files**

**File Name:** Supplementary Data 1

**Description:** Data used to derive Figures 1-7 in the main manuscript
